# The Executive Branch decisions in Brazil: A study of administrative decrees through machine learning and network analysis

**DOI:** 10.1371/journal.pone.0271741

**Published:** 2022-07-21

**Authors:** André Luís Ribeiro, Othávio Ruddá Araújo, Leonardo B. Oliveira, Magna Inácio

**Affiliations:** 1 Computer Science Department, Federal University of Minas Gerais, Belo Horizonte, Brazil; 2 Political Science Department, Federal University of Minas Gerais, Belo Horizonte, Brazil; Universiteit Maastricht, NETHERLANDS

## Abstract

This paper dissects the potential of state-of-the-art computational analysis to promote the investigation of government’s administrative decisions and politics. The Executive Branch generates massive amounts of textual data comprising daily decisions in several levels and stages of the law and decree-making processes. The use of automated text analysis to explore this data based on the substantive interests of scholars runs into computational challenges. Computational methods have been applied to texts from the Legislative and Judicial Branches; however, there barely are suitable taxonomies to automate the classification and analysis of the Executive’s administrative decrees. To solve this problem, we put forward a computational framework to analyze the Brazilian administrative decrees from 2000 to 2019. Our strategy to uncover the contents and patterns of the presidential decree-making is developed in three main steps. First, we conduct an unsupervised text analysis through the LDA algorithm for topic modeling. Second, building upon the LDA results, we propose two taxonomies for the classification of decrees: (a) the ministerial coauthorship of the decrees to map policy areas and (b) the decrees’ fields of law based on a tagging system provided by the Brazilian Senate. Using these taxonomies, we compare the performance of three supervised text classification algorithms: SVM, Convolutional Neural Network, and Hierarchical Attention Network, achieving F1-scores of up to 80% when automatically classifying decrees. Third, we analyze the network generated by links between decrees through centrality and clustering approaches, distinguishing a set of administrative decisions related to the president’s priorities in the economic policy area. Our findings confirm the potential of our computational framework to explore N-large datasets, advance exploratory studies, and generate testable propositions in different research areas. They advance the monitoring of Brazil’s administrative decree-making process that is shaped by the president’s priorities and by the interplay among cabinet members.

## Introduction

Administrative decrees are documents that manifest the Executive Branch decisions in Brazil. Similar to the United States’ executive orders, those documents are responsible for executing laws, implementing government plans, controlling public expenditure, and giving administrative directives to federal bureaucracies. These decrees are especially relevant in Latin American presidentialism, where the chief executive is vested with broader legislative and administrative powers. However, legal and constitutional restrictions on presidential discretion in issuing these decrees may vary the types of decisions and policy areas that presidents can target through these decrees. In some countries, such as Brazil, presidential discretion is broadened by the power to issue, in addition to executing law decrees, autonomous decrees, which do not require prior legislation and give greater latitude to presidential policymaking. In others, decrees are powerful tools for the distribution of individual benefits, as in Paraguay, Chile, and Peru. This massive collection of writings is the one of unilateral decisions of the president, capable of promoting important changes in public policies as well as impacting millions of lives and companies.

What are the decisions and policy areas most targeted by administrative decrees issued by Brazilian presidents? Is it possible to identify priority policy issues or areas in each administration based on thematic clustering, co-authorship, and inter-connectivity of the decrees? The prerogative of issuing decrees is a multi-targeting tool, but we know very little about the political issues and policy areas that the president and the cabinet members influence through this tool. Discriminating and classifying the content of these decisions is a necessary step to advance our understanding of presidential decree-making. Yet this is not a simple task. First and foremost, there is no automated tool to monitor and notify once new decrees are posted. Second, the government publishes decrees through a rudimentary IT system that does not provide, for instance, advanced search, recommendation, or download. Third, there barely is summarizing tools aiming to provide the user with an overview of each decree. Consequently, stakeholders must spend both time and capital to analyze the Executive Branch’s directives and their impacts.

Computational tools’ advances represent an opportunity to fix the problems above. Nowadays, one of the most promising areas of computer science is machine learning. The knowledge given by this area enables us to recognize and reproduce patterns in data. Particularly, machine learning may be applied for topic exploration and modeling [1], and for text classification [2–4]. On the analytical field, another prominent area is the one of complex networks, which allows researchers to get insights about the relational aspects of their object of study [5].

Computational tools also pave the way to a new era of technological innovation in the realm of politics. In fact, current computational tools act as a novel utility belt to evaluate and validate long-term theories [6] in the field of Political Science. They also facilitate the citizens’ access to federal government decisions and enhance the public control of the executive direct actions. For example, automatic text classification has been used in the Brazilian Judicial Branch to speed up the categorization of legal decisions [4]. Over the globe, in turn, automatic text classification has been employed in the Legislative Branches to boost efficiency and transparency [7–9].

Regarding the Executive Branch, on the other hand, still few works present computational results applied to direct acts of the president [10]. However, computational analysis is a promising approach to explore documents in an executive-centric system [6], highly dependent on presidential decisions, as Brazil. With this work, we aim to illuminate some of the aspects of the executive policy-making in the Federal Administration in Brazil through the computational analysis of administrative decrees.

In this work, we introduce a framework for analyzing Brazil’s administrative decrees at the federal level. Notably, we work over decrees of the last two decades in Brazil and:

- autonomously collect decrees via crawlers;
- explore the textual patterns of decrees based on an analysis of topics;
- automatically classify decrees based on two standard taxonomies;
- analyze decrees from a complex network perspective.

Given the collected documents, we begin by observing patterns of text through an algorithm of topic modeling. To do so, we made use of the Latent Dirichlet Allocation (LDA) model [11], a statistically-based unsupervised technique to analyze the tendency of documents of being topically related.

As for classification, we presented and reproduced a taxonomy based on the ministerial portfolios whose ministers sign each of our texts; and another taxonomy based on a tagging system of legal norms provided by the Brazilian Federal Senate. We addressed these classifications above using three different machine learning approaches. (1) A combination of *tf-idf* and Supporting Vector Machines (SVM). (2) A convolutional neural network. (3) A hierarchical neural network with an attention mechanism. We also combined the outputs of those three methods with a majority voting classifier.

Finally, for complex network analysis, we made use of graph theory to track the relevance, understand the organization, and assess strategic areas of administrative decrees. Precisely, we defined a directed graph with the nodes representing decrees and edges representing citations between decrees. Then, we explored this graph from the microscopic and mesoscopic levels, analyzing node centralities and also some of the clusters existent in the network. Based on the proposed taxonomies, we indicate what areas play central roles in the network.

### Background

In this section, we present the main concepts necessary for a better understanding of this work. We begin by describing some of the areas related to computer science that we made use of; then, we overview the methods used for topic modeling and document classification.

#### Machine learning

Traditionally, computers are manually programmed to perform specific tasks. That takes a human to analyze the context, find a solution, and implement an algorithm to solve it using the machine. But given an input, are computers capable of “learning”? Machine learning is the area of algorithms that deals with this question. It can be briefly defined as computational methods that, given the previous experience, can improve their own performance on a task or make accurate predictions [12].

Notably, we employ two learning approaches in this work: supervised and unsupervised learning. Suppose we know precisely the output for a given input. We could then use this preliminary information to conceive a model that reproduces this behavior for unseen inputs. This approach is the so-called *supervised learning* [13]. Note that this method is distinct from just memorizing the pair input/output (a problem called over-fitting), as we want our model to generalize for unseen data. Now suppose we no longer have any information regarding what is the expected output from a given input. In this case, in contrast, our learning is no longer assisted—or supervised—by some prior knowledge. This approach is therefore called *unsupervised learning* [13]. Finally, note that unsupervised learning is instrumental in inferring insights on data’s nature. And supervised learning is effective when one already possesses some understanding of the behavior to be reproduced.

#### Natural Language Processing (NLP)

Natural Languages, such as English and Portuguese, are the way used by humans every day to communicate with each other. The computers simulate an intelligence able to “understand” natural languages through algorithms by reading and interpreting word sequences. This process is called NLP [14].

The process of understanding human-computer utterance through NLP may occur with several statistical models as defended by Christopher Manning and Hinrich Schutze [15]. Although there are many NLP models, the processing of natural language is a wide area of computer science with recent models. As the relation between humans and computers grows, and machine learning advances, NLP evolves with increasingly complex and useful applications.

#### Unsupervised learning for topic modeling

Unsupervised learning is an area of machine learning used to deal with unlabeled data or data of *unknown* structure, as described by Sebastian Raschka [1]. Techniques of unsupervised learning are, usually, applied to find subgroups with clustering given their capacity of exploring data structure and of extracting meaningful information without any kind of label guidance.

In topic modeling, the data is provided to the model after a pre-processing step as done by Jacobi et al. [16]. First, the data is converted in a tokenized structure, usually a list of words. Second, in order to avoid different words with similar meanings, it is possible to include a lemmatization step, i.e, the words are reduced to a primitive form. For example, the words *weakness* and *weakly* become, simply, *weak*.

The topic modeling with LDA [11] produces topics where each component is represented by a distribution over words. To do so, LDA uses an efficient process based on a probabilistic Bayesian model which provides an explicit group representation of related words that can be visualized by a tool called LDAvis.

The LDAvis tool shows a 2-D space, known as the *Intertopic Distance Map*, with circular representations of topics whose distances and centers are determined using multidimensional scaling [17]. Each axis of LDAvis is unrepresentative by itself due to the dimensionality reduction, however, the areas, positions, and distances of the topics provide a valuable representation of the topics. The area of each circle is proportional to the prevalence of each topic in the entire corpus. LDAvis also shows a bar chart with the content of each topic according to the parameter λ. The λ is a weighting parameter that determines the way that the terms are sorted in the bar chart. By setting λ close to 0, the terms are sorted decreasingly by the relevance average that considers the ratio between the estimated term frequency within the topic (represented as the red bars) and the overall frequency of the term in the whole corpus (represented as the blue bars). On the other hand, by setting λ close to 1, the terms are sorted mainly by the estimated term frequency within the topic, also in decreasing order.

#### Supervised learning for document classification

**Fig 1:**
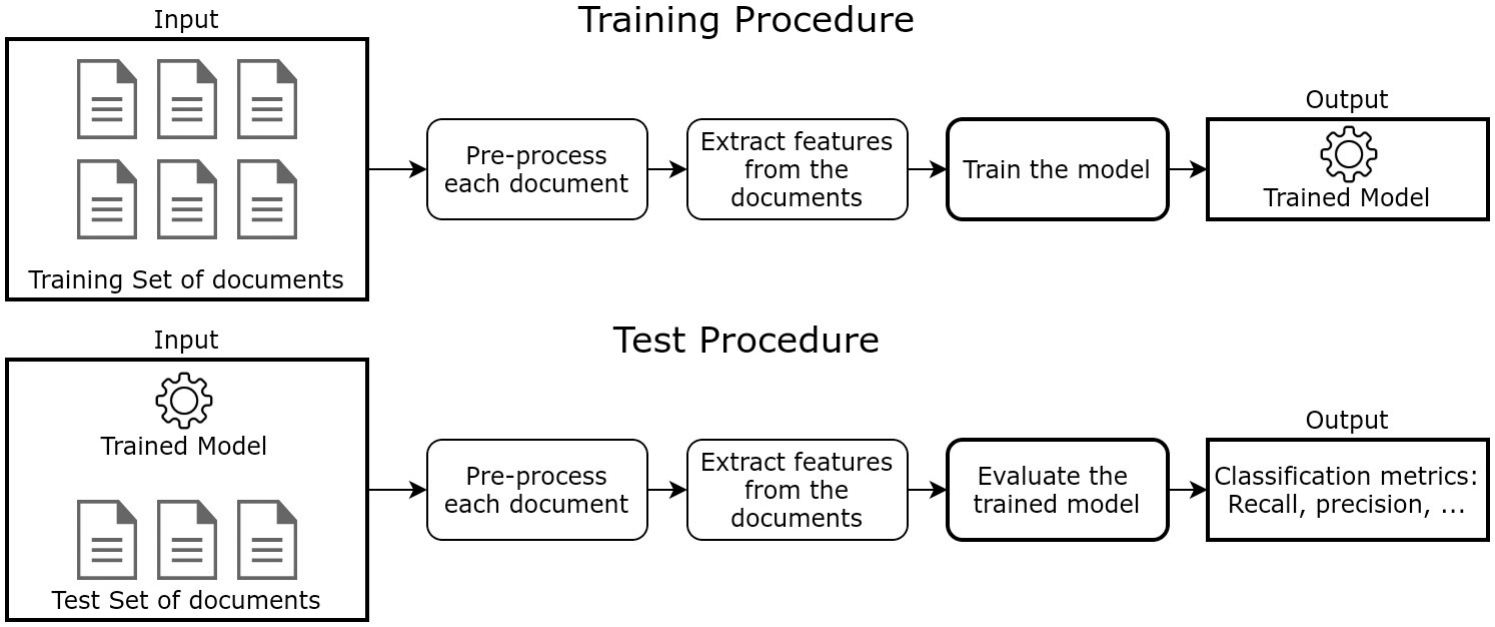
Supervised learning for document classification.

In supervised learning, classification refers to assigning a category to each item [12]. Classification problems are (1) mono-label multiclass, i.e., two or more classes and each item getting only one label; or (2) multi-label multiclass, i.e., two or more classes, and each item getting two or more labels. Regardless of the type, document classification requires first obtaining numerical representations for text and training a classifier. A simplification of the standard process is presented in Fig 1, based on [18].

The process has two parts: training and testing. When dealing with supervised learning, it is crucial to test the models on samples other than those from the training set, as the training optimizes the results for its own collection. Therefore, one more set, the test one, is kept apart from the first and used for the final evaluation of the model.

Both training and testing also include pre-processing. Pre-processing often includes removing stopwords (recurrent vocables that may not contribute to the classification like“the”, “a”), applying lemmatization, and normalizing document length. Approaches like *tf-idf* and embeddings are commonly used for textual representations, whereas classification strategies like SVMs are generally employed for modeling.

In this work, we explore *tf-idf* in tandem with SVM as well as word embeddings together with convolutional and recurrent neural networks for decrees classification. We adopted this approach because *tf-idf* combined with SVMs [2, 19] and certain neural network architectures are known to perform text classification accurately [20].

#### Metrics for evaluating classification models

To evaluate a supervised learning performance in a classification task, it is possible to measure the model quality through metrics like precision, recall, and f1-score. Each metric represents a different statistical view and it is used in different contexts. Recall and precision relate to how much of a given class *c* was actually predicted as *c* and, of the amount predicted as *c*, how much was actual *c*, respectively. Informally, high recall means that no relevant samples were left out, whereas high precision implies “no garbage” has been collected. Those metrics, especially recall and precision, often pose trade-offs; namely, one’s optimization is detrimental to the other. On the other hand, f1-score (or f-measure) is a metric that signifies the harmonic mean of recall and precision, which means that both recall and precision must get higher in order to increase the overall f1-score.

An aggregate measure is usually applied when the algorithm deals with multiclass tasks [20]. For that, two possibilities are the macro and micro averages. The macro average gives a simple average over the results gotten from each class. Unlike the macro average, which is considered to be a per-class average, the micro average assigns equal weights to every document. The micro average is, therefore, considered to be a per-document average.

## Related work

In this section, we explore some of the existing work on text and network analysis. For each topic, we begin by briefly presenting some overall works in the area. We then discuss similar works in the realm of political science. Finally, we discuss what has been done in the Brazilian context, specifically. Because few studies have focused on the Executive Branch, especially Brazil’s, we included works done in the other branches or countries. The overview of what has been done in the other branches and countries allowed us to define what algorithms and methodologies should be explored by this work and what should be the expected performance.

### Document classification and topic modeling

The longitudinal and large N-data of presidents’ administrative decisions enable researchers to uncover how the government is driving policies, executing laws, and allocating budget resources. However, these administrative decisions’ volume and complexity have inhibited their regular use by political scientists, legislators, lawyers, experts, and whoever is affected by these decrees. Since 1990, more than ten thousand decrees have been issued by Brazilian presidents. Tools for automated text methods have been increasingly applied to overcome these limits. According to the researcher’s substantive interests, these decisions’ complexity requires more sophisticated text-as-data methods for estimating valid measures. Such methods are crucial to understanding presidential policy-making, especially in Brazil since decrees do not identify their respective policy areas.

Topic modeling is a relevant NLP approach to extract information out of a textual dataset in an unsupervised manner. LDA is one of the algorithms that can be used to perform this task, however its results depend on the amount of text given to the algorithm. Specifically in the case of LDA, samples with short texts, e.g. tweets [21], can lead to less coherent results than samples with long texts. Therefore, some works discuss different procedures to outperform traditional LDA specifically in short sample cases [22, 23]. Another possible approach to deal with short texts is to merge micro texts into longer texts before performing the topic modeling, as done by Mehrotra et al. [24] for tweets. The discussion of how the input of the LDA affects its coherence becomes particularly relevant in cases like ours, in which there are two representations of the data: a short abstract with denser information and the full text of the administrative decree (see Materials and methods).

Another approach to explore massive textual content is through text classification. Text classification has already been explored in the legal domain. Sulea et al. [7] develop an SVM ensemble for predicting the law area of cases and rulings from a collection of documents from the French Supreme Court. Their work reports 96.6% of f1-score in this task, focusing on the benefits of automatic text classification to support law professionals’ decisions.

Also in the Judicial Branch, Undavia et al. [9] discuss some approaches with neural networks for text classification in a United States Supreme Court dataset. The authors also explore taxonomies, similarly to what we have proposed for administrative decrees. In their case, two taxonomies were explored: one broader with 15 classes, similar to ours, and another with finer-grained classes, with more than 250 labels. Their convolutional neural network (CNN) presents results with more than two times accuracy over the broader taxonomies than over the fine-grained. For the broader, the authors report 72.4% of accuracy with the CNN, while for the fine-grained, 31.9%. In our case, instead of exploring the effects of different granularities, we explored the differences between two taxonomies with different focuses.

As for the Legislative Branch, O’Neil et al. [25] applied topic modeling in legal texts of the United Kingdom. To tune the LDA parameter in a fair manner, they counted on a legal practitioner that manually assessed their models. The legal practitioner was in charge of defining the number of topics of the LDA and assisting in the definition of the main parameter of the LDAvis [26] visualization tool. Their approach evidence how computational methods should be combined with the specific knowledge from the research area in order to get comprehensive insights about the constitution of the data explored through topic modeling. In turn, Chalkidis et al. [8] present a comparison of approaches for classification of the European Union documents. Their work tends to an extreme multi-label text classification task, as they have dealt with thousands of labels, some of them also with few or zero samples in the test set. They tested many approaches, including HAN, a convolutional network, and some other models like BERT [27]. Including all labels, their best result was given by BERT, with 73.2% of micro-f1.

Regarding the Executive Branch, Ruhl et al. [10] discuss topic modeling for analyzing a collection of executive orders from the United States of America. They manually explore the coherence and semantic meaning of the generated topics. For extensive collections of texts, they also advocate for machine learning in the legal domain as tools to accelerate analysis and give insights about the content of documents, though still not replacing humans, only facilitating some processes. Kaufman [6] in turn presents potential applications of machine learning in the future of political science analysis in the Executive Branch of the United States.

As the language particularities are factors that can influence many textual approaches, it is important to also take a look at what has been done in Portuguese, especially in Brazil and for texts from the political science domain. In Brazil, most of the work applying computational methods to political science documents has been done in the Judicial Branch. Luz de Araujo et al. [4] present the Brazilian Supreme Court database and several approaches for automatic classification in this domain, including an SVM, an LSTM, and a CNN. On their medium dataset, the best result for document classification is given by their LSTM, with 70.92% macro-f1. Da Silva et al. [28] address the same problem, focusing on the primary studies performed with a standard convolutional neural network for text classification that has gotten 91% of f1-score in a small dataset. Both works refer to the Supreme Court’s initiative to speed up the internal process of text categorization in the courts of Brazil.

Finally, using structural topic modeling (STM) for an unsupervised machine learning model, Inácio and Recch [29] identified and compared major policy areas targeted by Brazilian presidents. Notable differences in the prevalence of topics reveal that presidential attention varies considerably when presidents take unilateral actions by issuing decrees. While some presidents issued plenty of decrees to implement social policies, others prioritized changes in regulatory policies and state reforms.

### Complex networks

The analysis of complex networks has also been employed in many domains with the intentions of characterizing the structure created by links in a collection, exploring specific groups, and finding important individuals. In the context of information retrieval, for example, the web has been represented as a graph by Broder et al. [30]. This representation allowed the authors to explore this network’s formation from different perspectives, from micro to macro levels. In the political science realm, models based on graphs also have been extensively used. Ward et al. [5], for instance, present some of those applications and the available methods for network exploration, indicating the possibilities and advances for mapping the relations between individuals and institutions from the political environment.

Networks have been used to describe the relations among decisions described in political documents. In New Zealand, records from the Legislative Branch have been modeled through a network [31]. Similarly, Bommarito et al. [32] discuss different perspectives of the networks generated by the courts’ documents of the United States. In turn, Mazzega et al. [33] present the structure of French legal codes based on the existing citations between those texts. Based on that, the authors also notice a rich club of documents, which tend to be more cited and therefore may perform a more significant role.

Also, Koniaris et al. [34] describe the citation network comprising legal texts from the European Union, focusing on the macro aspects of this network. The authors observe general elements of this structure and relate it with the network formation throughout time. Also, a parallel can be drawn between the macroscopic properties of this network and the one presented by Bommarito et al. [32], as both networks are well represented by a bow-tie decomposition. We did not find any specific work in Brazil applying complex networks’ approaches to model collections of administrative decrees. Table 1 summarizes the works selected in this section.

**Table 1:** Related work.

Work	Branch	Models	Locale
Zhao et al. (2011) [21]	-	LDA	-
Yan et al. (2013) [23]	-	Biterm Topic Modeling	-
Mehrotra et al. (2013) [24]	-	LDA	-
Mazarura and De Waal (2016) [22]	-	LDA (modified)	-
O’Neil et al. (2016) [25]	Legislative	LDA	U.K.
Sulea et al. (2017) [7]	Judicial	SVM Ensemble	France
Da Silva et al. (2018) [28]	Judicial	CNN	Brazil
Ruhl et al. (2018) [10]	Executive	Conventional/computational topic models	U.S.
Undavia et al. (2018) [9]	Judicial	CNN, GRU, LSTM, Logistic Regression	U.S.
Chalkidis et al. (2019) [8]	Legislative	HAN, GRU (various), CNN, BERT, …	E.U.
Inácio and Recch (2019) [29]	Executive	STM	Brazil
Kaufman (2020) [6]	Executive	Topic models (LDA/variants)	U.S.
Luz de Araujo et al. (2020) [4]	Judicial	Naive Bayes, SVM, LSTM, CNN, …	Brazil
Bommarito et al. (2009) [32]	Judicial	Complex network	U.S.
Mazzega et al. (2009) [33]	Legislative	Complex network	France
Broder et al. (2011) [30]	-	Complex network	World
Ward et al. (2011) [5]	Overall	Complex network	-
Sakhaee et al. (2016) [31]	Legislative	Complex network	N.Z.
Koniaris et al. (2018) [34]	Legislative	Complex network	E.U.
This work	Executive	SVM, CNN, HAN, LDA, Complex network	Brazil

## Materials and methods

Although the president is the only authority with the power to issue decrees, the decree-making process involves the ministers in charge of the respective policy areas. In general, these ministers co-author drafts of the decrees for the president’s final word. These decrees can be signed by the president alone or with ministers from the corresponding policy areas.

The Brazilian Presidency has no formal mechanism for cabinet decision-making; however, analysis of the co-authorship of decrees allows us to uncover essential patterns of policy networks within the presidential cabinet. Given the informal gatekeeper powers of some ministries over executive decisions, regarding their budgetary or legal effects, the co-signing of decrees by Ministers of Finance, Planning, Justice, and Chief of Staff is frequent. Nevertheless, presidents have issued decrees on their own, particularly in cases of intra-cabinet divergences. Consequently, the signing of administrative decrees is of substantive interest to researchers looking at the cabinet decision-making process.

**Fig 2:**
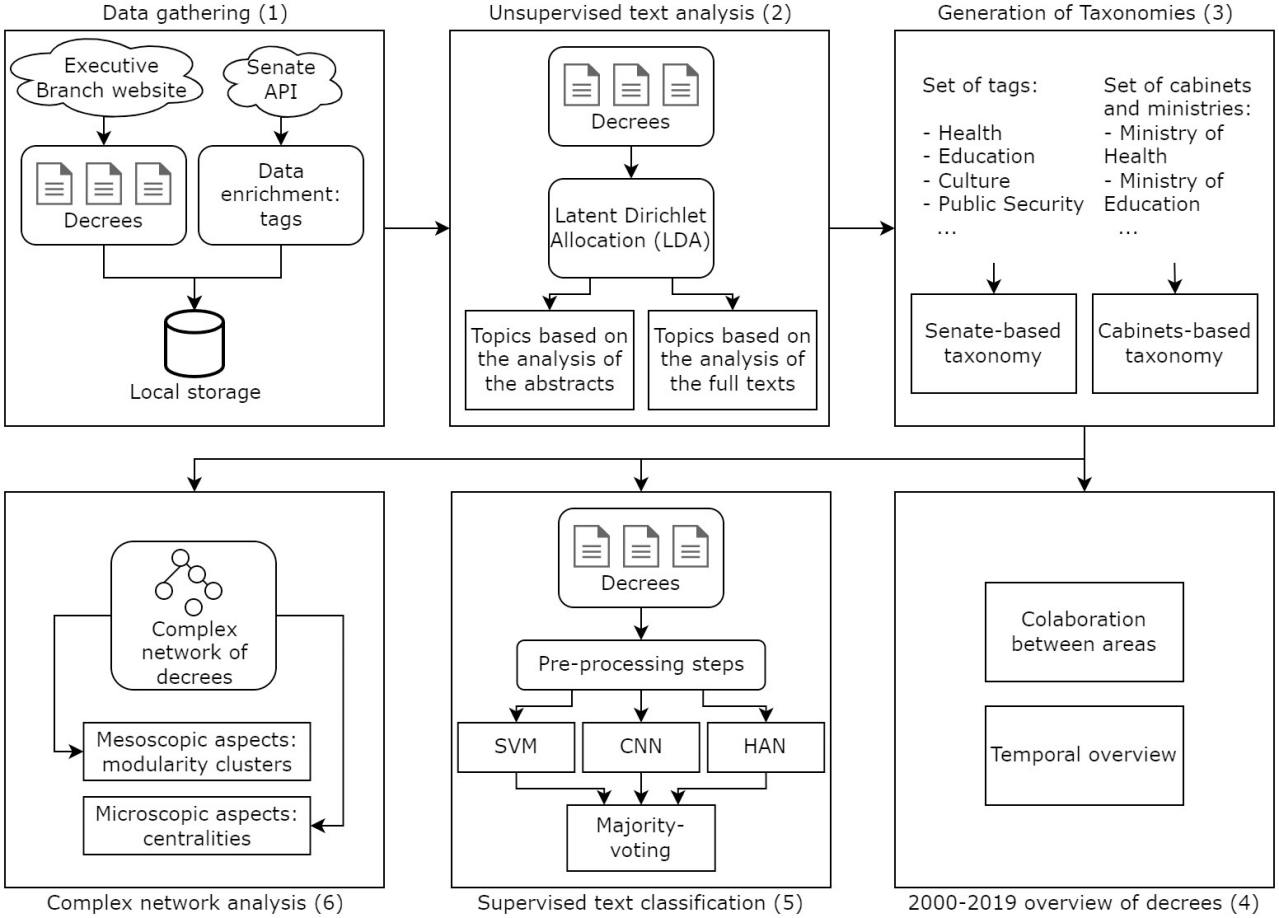
The framework for analysis of administrative decrees.

In this work, we have assessed the numbered administrative decrees from 2000 to 2019 in Brazil. Those are publicly available in the Executive Branch website (http://www.planalto.gov.br/CCIVIL_03/decreto/_Dec_ano.htm—accessed on April 23, 2022) or in the Official Gazette of the Federal Government in Brazil called *The Diário Oficial da União* (DOU). The overall framework used in this paper is summarized in Fig 2. We have gone through all the process of collecting and analyzing Brazilian administrative decrees from different perspectives.

We begin by systematically collecting those decrees from the website (Fig 2(1)). At the end of this process, we ended up with a collection of 6,841 documents. Brazil had five presidents during this period, and each of the decrees is signed by the corresponding president. In exceptional situations, the main signature was given by the vice-president. Each decree is linked to a table with other details about the text, including the date it was signed, the president who has signed it, whether it was later revoked or not, the related cabinets and areas, the number of the act, and its origin. Finally, each decree presents an abstract that summarizes its impact in the area it affects. S1 File reproduces the structure of an online decree together with the table linked to that decree.

The decrees of the collection relate to 181 different ministries and agencies. This wide variety of names is due to differences in the organization of the Executive structure over the years and acronyms/terms that are not adequately standardized.

### Data gathering and standard pre-processing

Decree links are grouped by year on the Executive Branch website. We accessed each of those links and crawled the decrees. As usual in static pages on the web, the HTML pattern available at each year page was not standard; therefore, manual fixes had to be done on the crawling algorithm.

We used web scraping to obtain and filter the data available at decrees pages automatically. The terms and conditions for the website applied while the decrees were extracted are detailed by Ordinance number 1,492 from October 5, 2011 (see http://www.planalto.gov.br/ccivil_03/Portaria/P1492-11-ccivil.htm—accessed on April 23, 2022). This ordinance defines that the use of the platform is granted for sharing and creating non-profit derivatives, provided the source and the date of access and not including national symbols. No specific extraction method is interposed by those terms. As specified by them, we also state that the texts presented in the website and extracted for this work have only informational purposes.

Our main goal while crawling those pages was to extract the full text of the decree and its abstract, loading it on the local database. We also removed all appendixes from the final text during this process and saved every HTML anchor link available in the document. Finally, we focused on obtaining the related ministries, decree identifications, and the president who has signed the decree for each table. All this data has been structured in an SQLite database.

Before running any machine learning technique over decrees, we applied a tailor-made pre-processing that removes signatures at the end of each document, lower-cases texts, removes special non-Latin characters, and standardizes punctuation. Signatures are removed to ensure that signatories of decrees do not influence classifications and insights given by the models.

### Data enrichment

Though the table of each decree already presents a tagging system (in this paper, we refer to tags as the terms that are used to describe the decrees on the original databases, and to labels as the descriptions given after the creation of our taxonomies) based on subjects and law fields, these tags present low granularity, with thousands of unique terms for our collection of decrees. Therefore, searching or classifying decrees through these tags is challenging and involves dealing with highly rare or ambiguous terms. Instead, we opted to enrich the dataset with tags provided by other instances of the government.

In Brazil, the Legislative Branch plays a primary role in gathering all data from legal documents, including the Executive Branch documents. The Brazilian Senate presents an Application Programming Interface (API) that includes information from administrative decrees, with another tagging (labeling) system that deals with fewer tags (approximately 500) and more aggregated information. Using the API calls, we matched the Senate tags with each of the collected decrees and proposed a new taxonomy based on these tags named *Senate-based taxonomy*.

### Unsupervised text analysis with LDA

We applied topic modeling in two distinct representations of each decree using a LDA model. First, we have explored the behavior of this model only with the abstract of each decree, a less specific representation, but with denser information. Second, we have worked with the concatenation of the abstract with the decree’s full text, a representation with wider information. Moreover, we have defined the number of topics for each approach using the best parameter selection search with the Grid Search algorithm, the method discussed by Feurer et al. [35]. Grid Search constructs multiple LDA models for all possible combinations of parameter values and compares them until finding the highest set of parameter values. In this case, we have maximized the statistical model log-likelihood, which measures the coherence of unseen data with data already processed [11], to evaluate the similarity between topics.

After the LDA process, we have used the LDAvis. In this step, we have searched for the best presentation of the data that shows a meaningful list of terms in the topic. Therefore, we have set the optimal λ to 0.4 through a case study observing the results given by different λ across all topics and labeling each one manually.

### Taxonomies

Based on the unsupervised experiments’ insights and on the tags and information already available and gathered on our database, we present two taxonomies for the classification of administrative decrees.

The first is the cabinet-based taxonomy, which is based on the nomenclature of the ministerial portfolios in each administration at the federal level of government. The identification of those portfolios is based on the ministers who sign each decree. The second taxonomy is based on groups of tags, related to the fields of law, provided by the Senate API.

Both taxonomies present high granularity. It is worth noting that we can increase sampling and cope with changes in the Executive organization by grouping related areas, including nominal and cabinet portfolio changes. However, the flip side is that we lose the specificity of each area.

**Fig 3:**
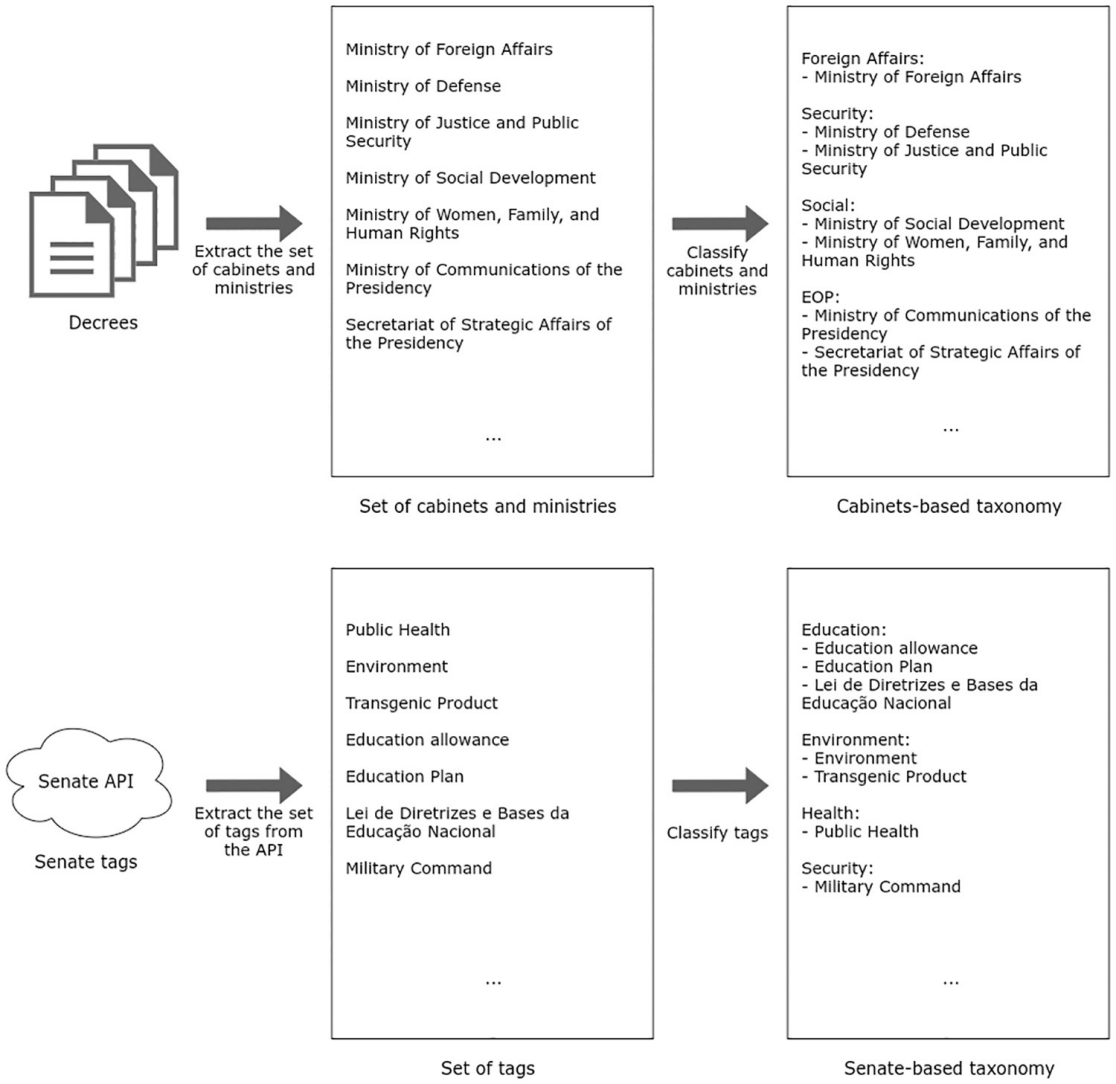
Process of creation of taxonomies.

The creation of the taxonomies is illustrated in Fig 3. Basically, we have manually organized the set of ministerial portfolios in groups. The same process was performed for the Senate-based taxonomy, but in that case, using the set of the Senate tags.

The process of creation of taxonomies considered the composition of the presidential cabinet over time; the fields of law in each decree; and the topics identified in the analysis of the LDA. Therefore, we consider the LDA model an exploratory step to get a full comprehension of the administrative decrees’ dataset before creating the taxonomies. An important caveat is that we do not compare LDA models with the two taxonomies, as their nature and methodologies are different.

### Supervised text classification

The organization of administrative decrees in taxonomy groups provides a simple but relevant indicator of the actions and priorities of the Executive Branch over time. However, maintaining the entire Brazilian database updated with those indicators is a process that takes both time and money. In this context, supervised machine learning techniques can provide mechanisms to reproduce those taxonomies for new decrees. The models generated by those techniques allow us to immediately infer which areas are affected mainly by the recent acts of the Executive and also provide a consistent point of view between different time changes in the organization of cabinets, portfolios, and themes.

The challenge is this: given a set of decrees and their classes, can we reproduce the logical mechanisms that link those decrees to their taxonomies? To address that, we made use of three well-established supervised learning techniques. One is based on SVM [2], while the other two are based on neural networks, respectively, a convolutional neural network inspired in that of [28] and a hierarchical neural network, designed for document classification [3]. We also ensemble the results of those three methods in a majority voting classifier. Besides, we used both the abstract and the decree’s full text as input to the models, based on the results given by the LDA analysis.

To further test the quality of the models obtained, we begin by separating 20% of the data for evaluation. The rest of the data is broken into four other sets used for training and tuning the hyper-parameters of the models based on a 4-fold-cross validation. For hyper-parameter tuning, we made use of Bayesian optimization for efficiently searching in wide spaces of parameters. For that, each of the four sets was used as validation for a model trained with the other three. The mean of the macro F1-score across these models was used as the metric to evaluate the performance of the hyper-parameters in each iteration of the optimization.

The technique’s approach and classes’ distributions are described in S2 File, together with the distributions of the classes of each set and with the best hyper-parameters found during optimization. Broadly, we used the same stratified sets for all techniques. We also applied pre-processing methods to those decrees according to what we found most appropriate based on the literature of each approach.

### Network analysis

We also have modeled the collection of decrees as a graph. We begin by creating a set of nodes *N* with all decrees of our collection. Then, given a node *a* representing a decree *D*_*a*_ and a node *b* representing a decree *D*_*b*_, a directed edge exists from *b* to *a* if and only if *D*_*a*_ was published earlier than *D*_*b*_ and *D*_*b*_ has a link to *D*_*a*_. Links are obtained directly from the HTML of each decree. The set of pairs (*b*, *a*) originates the set of directed edges, *E*_*d*_, of our graph. Finally, we keep only nodes with one or more incident edges to them. The final graph is then given by *G*_*d*_(*N*, *E*_*d*_).

The first level of analysis in this graph is made by looking at the most “central” nodes. Different centrality measures have been used to estimate the importance of each node of a network, but two simple varieties are the *degree* centrality and the *betweenness* centrality. The degree of a node is given by the number of nodes adjacent to it. In our case, as we have modeled a directed graph, we give degree centrality by the sum of in-degree (edges coming into the node) and out-degree (edges coming out of a node) of a node. So, in practice, degree centrality indicates how much nodes are connected to other nodes. To compute betweenness, in turn, one uses all the shortest paths between any two nodes that include the target one and outputs an indicator of how much the network structure would be affected if the target node had been removed. For more details on betweenness and degree centralities, please refer to [36].

The second level of analysis is performed by exploring the mesoscopic structure of our network through the perspective of modularity classes. Modularity indicates community structures in a network [37]. Particularly, we made use of the algorithm given by [38], implemented on Gephi (see Gephi 0.9.2 visualization and exploration software: https://gephi.org/—accessed on November 20, 2021), to separate our network into clusters. Then, we analyze the structure given by those clusters from our taxonomies’ perspective. We do not perform any specific parameter tuning during this analysis (the results were obtained using resolution 1.0 and with randomness by default), and we emphasize that the approach implemented by Gephi is a heuristic with results that can vary due to randomness. Hence, we face these results from an analytical perspective, with the primary intention of finding and exploring specific regions of the graph, and also suggest further studies in this direction.

## Results and discussion

### Unsupervised text analysis with LDA

In this section, we present the results of the LDA algorithm in two different analyses. First, we have provided only the abstract of the decrees as an input to the algorithm. Then, in the second analysis, we have provided to LDA the entire content of the decrees.

#### Decree’s abstract analysis

**Fig 4:**
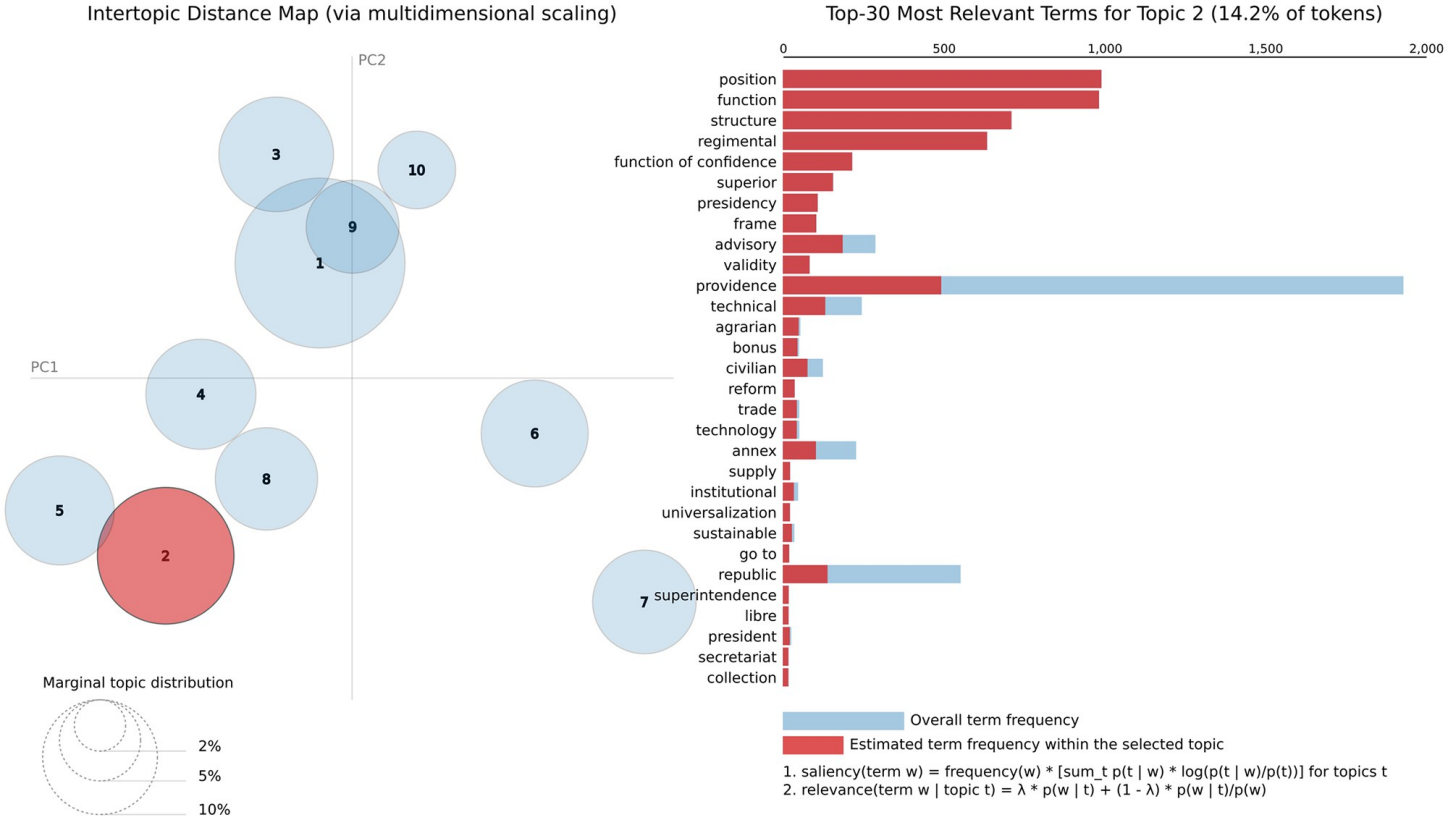
LDAvis results for abstract only.

Fig 4 illustrates the results of the abstract analysis with 10 topics. In this case, we opted to show, as an example, the top-30 most relevant terms for Topic 2. For an overall visualization of these results, Table 2 details each of the 10 estimated topics, with the top-5 most relevant terms and a description of each topic.

**Table 2:** Top 5 LDAvis terms for abstract analysis using λ = 0.4.

Topic #	Top-5 most relevant terms	Description
1	enact, law, regulate, october, provision	regulatory decrees
2	position, function, structure, regimental, trust	civil services
3	federal, incident, tax, aliquot, product	taxes
4	public, federal, ambit, administration, alter	public management
5	social, national, provision, functioning, inclusion	civil services
6	govern, cooperation, international, convention, common	foreign affairs and concessions
7	economic, additional, protocol, complementation, republic	international economic agreements
8	financial, fiscal year, executive, budget, programming	budget allocation
9	drafting, new, competence, provide, assistance	administrative decrees
10	officiate, promotion, incidence, time, impose	militarism

Here, we briefly describe the main findings from this topic model. An example of near and correlated topics, with an overlapped area, is demonstrated by topics 1 and 9. They are easily detected in Fig 4. They both refer to terms related to the type of decision made. They discriminated different decisions, Topic 1 being more related to executive directives for the execution of laws and Topic 9 more associated with administrative orders.

Presidents issue this type of decree to put the executive branch into operation. Terms referring to the regulation of the civil service and giving directions for bureaucracies are captured by prevalent terms of topics 2 and 5, with the slightly overlapping circles. Topic 4 refers to the federal level of public administration in charge of managing budgetary resources and national policies. It is sided by Topic 8 which captures the budgetary decisions, which are critical to government tasks. It refers to budget allocation among federal administration units, but also inter-branches transfers or to sub-national governments. Interestingly, it is not correlated to topic 3, which alludes more to state funding; tax regulation, and collection, as depicted in the Map Fig 4.

One of the distinctly different clusters is formed by topics 6 and 7, isolated in the Map. The former comprise terms associated with foreign affairs and diplomatic issues, while the latter refers to commercial agreements and economic cooperation treaties. It is worth noticing that these documents have a very peculiar semantic structure, aligned with international laws, and they write by the diplomatic bureaucracies. Finally, topic 10 is relatively ambiguous, including military-related functional issues, but also nonspecific words.

As in this section we have worked with only the abstracts, which are short samples of text, we expected that the LDA would not be able to detect more specific topics, as well as illustrated by Zhao et al. [21]. Moreover, the abstract data does not precisely identify the theme, but rather a short description of the subject of the decrees. Therefore, we applied another analysis with the full decree.

#### Full decree analysis

**Fig 5:**
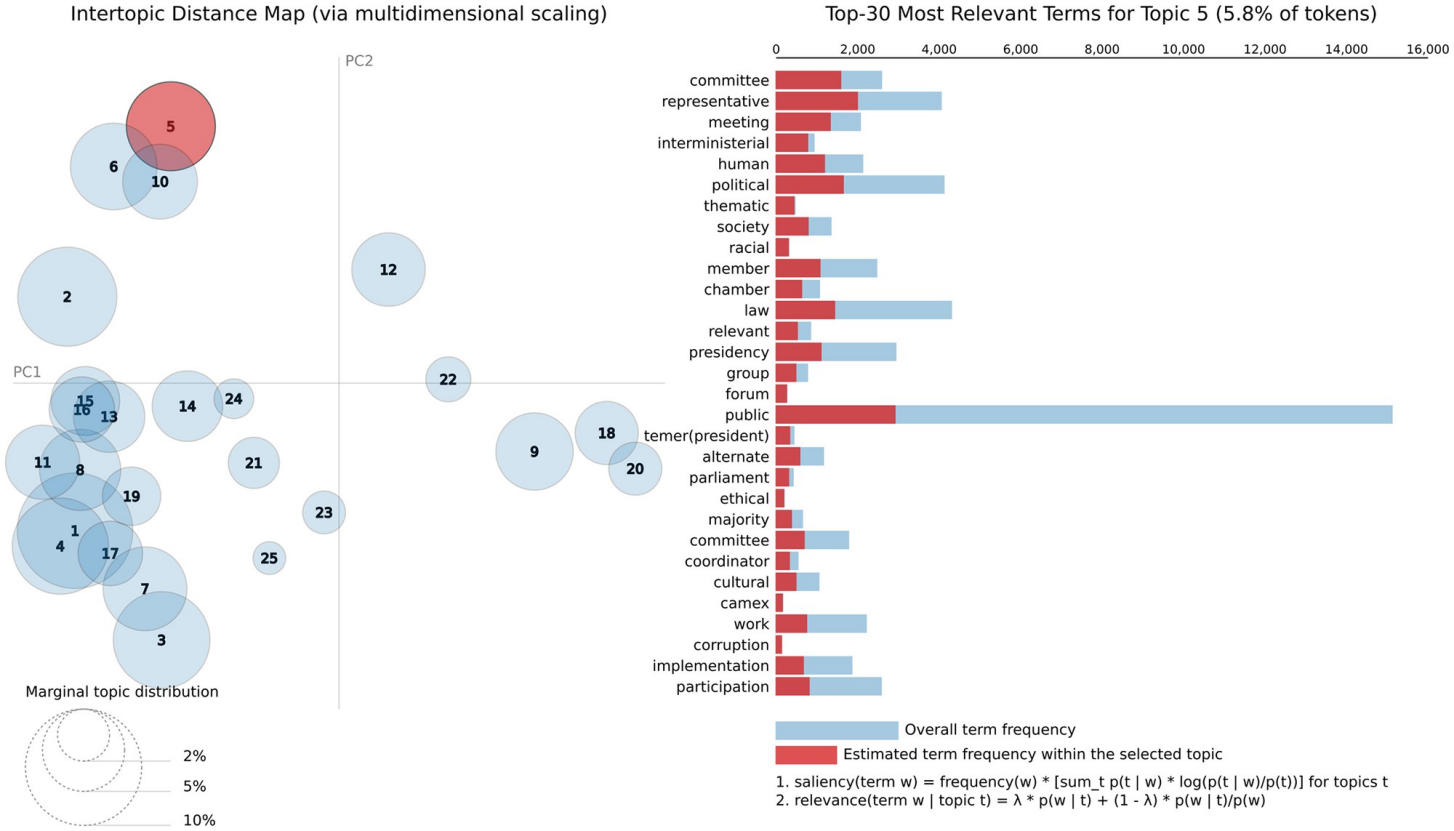
LDAvis results for full decree.

We present the results of the LDA with the full text of the decrees in Table 3, which discriminates the 25 estimated topics together with the top-5 most relevant terms and a thematic description of them. An overall view of the cluster of these estimated topics is depicted by Fig 5.

**Table 3:** Top 5 LDAvis terms for full decree using λ = 0.4.

Topic #	Top-5 most relevant terms	Description
1	deadline, decision, legal, hypothesis, request	government decisions
2	security, greeting, information, assistance, child	social assistance
3	goods, regimen, customs, import, rate	import regulations
4	product, agrochemical, packaging, fire, animal	enviroment and food products
5	commission, representative, meeting, interministerial, human	advisory committees
6	control, activities, executive, strategic, agency	positions and comissions
7	operation, value, tax, settlement, credit	taxes
8	rural, family, agrarian, property, area	agrarian policies
9	December, confer, January, May, September	non respresentative topic: dates
10	council, writing, member, representative, trade	sectoral commissions
11	price, contracting, bidding, preference, edict	government procurement
12	regimental, function, position, advisory, structure	personnel regulations
13	public servant, evaluation, perform, individual, score	performance evaluation of civil service
14	expense, financial, budget, appropriation, apostille	budget allocation
15	research, scientific, technological, development, innovation	technology and innovation
16	course, teach, professional, institution, education	education regulation
17	electric, energy, concessionaire, undertaking, transmission	electrical generation concessions
18	agree, govern, congress, convention, review	foreign agreements
19	military, arm, police, senior, official	defense and security
20	complementation, economic, federative, republic, protocol	national government agreements
21	corporate, state, expense, inss, social security	contributions and social security taxes
22	resolution, merit, medal, growth, privatization	honorary award
23	aeronautical, officiate, naval, tourist, body	military
24	port, capital, maritime, port, facility	ports system
25	pontar, line, follow, wine, grape	non representative topic: messy words

Different from the models with the abstracts, the models with the full text of the decrees discriminate the decisions of the Executive Branch in the same regulatory or administrative area. For example, Fig 5 presents the clusters formed by the topics, detailing for topic 5 the top-30 most relevant terms.

At the top of this figure, we can see the cluster formed by topics 5, 6, and 10, which have overlapping areas. They refer to the commissions ordinarily formed in support of the decision-making process within the Executive Branch and the agencies it regulates. However, the topics discriminate the advisory commissions linked to ministries (top 5) of sectoral collegiate bodies (top 10) and regulatory decisions of these structures (top 6). In the lower-left quadrant of the figure, we have a cluster formed by several topics related to the administrative activities of the Executive Branch. An example are the decisions on taxation and credits, differentiated by decisions on the taxation of imports (topic 3) and taxation of domestic activities and products. Finally, as observed in the analysis of the abstracts of the decrees, the topics related to decisions on foreign policy and international agreements (topics 18 and 20) are distant from the others in the intertopic distance map. As already noted, this distance reflects the boundaries between domestic policy and foreign policy, a recurrent trait of contemporary states.

Furthermore, a brief description of each topic is given in Table 3.

### Taxonomies

We present 13 classes for the cabinets-based taxonomy. Those classes focus on the organizational structure of the Executive Branch in Brazil during the time course of the database. A brief description of each is given in Table 4 that shows the main cabinets related to each class.

**Table 4:** Cabinets-based taxonomy description.

Class	Policy-areas
**Agriculture**	Agriculture, livestock, fishing cabinets
**Economy**	Economy, planning, farm, management ministries
**Education**	Education, culture, and sports cabinets
**Environment**	Environment cabinet
**Executive Office of the President (EOP)**	Presidency cabinets, institutional cabinets, strategic matters
**Foreign Affairs**	Foreign Affairs Cabinet, other diplomacy institutions
**Health**	Health ministry
**Industry**	Industries, cities, energy and mines, tourism and business ministries
**Labor**	Labor and foresight cabinets
**Science**	Science, technology, innovations, communications ministries
**Security**	Justice, public and institutional security, army
**Social**	Human rights, citizenship, social assistance, fighting hunger cabinets
**Transports**	Transports, ports, civil aviation

In turn, we present 15 classes for the Senate-based taxonomy (Table 5). Those classes focus on the legal effects resulting from each decree.

**Table 5:** Senate-based taxonomy description.

Class	Fields of law
**Agriculture**	Agricultural products and territories, food production, fishing
**Economy**	Financial system, public debts, government contracts
**Education**	Elementary school, technical and superior courses, culture, and sports
**Environment**	Environmental resources, forests, pollution, weather changes
**Executive Branch organization**	Administrative organization, positions and commissions, public assets, presidency
**Foreign Affairs**	International cooperation acts, diplomacy, foreign trade
**Health**	Public health system, hospitals, and health policy
**Industry**	National industry, infrastructure, transports, commercial activities
**Justice**	Civil rights, technical standards, regulations
**Labor**	Foresight and labor standards
**Mines and energy**	National energy matrix, mining
**Science**	Technology, communications, computing, data
**Security**	National and public security, including army, navy, aeronautics, and police
**Social**	Human rights, public assistance, land reform
**Taxes**	Tax breaks, taxation

These classes cover policy areas targeted by decrees already identified by the unsupervised analysis, for instance, defense, foreign affairs, and economic regulation. Decrees related to these salient policy areas reveal great text consistency and a tendency to form clusters, as demonstrated by LDA. However, presidential decree-making in Brazil encompasses a variety of policies and administrative decisions. In addition, cross-cutting issues are regulated by different executive agencies, and their relevance reflects new legislation or changes in government agendas and priorities. For instance, culture- and sports-related policies have been implemented by the Minister of Education or by specific portfolios created to manage these policies. Thus, both taxonomies reflect and categorize central issues driving the issuance of administrative decrees across several administrations.

However, these two taxonomies differ in their concepts and purposes. The cabinets-based taxonomy reflects how ministries’ jurisdictions are set up in each administration. I.e., the policy areas and administrative tasks in charge of each ministry. It reveals presidents’ choices regarding the size and design of the cabinet. For example, while one president implements social security and welfare policies through two different ministries, others prefer to merge them into a single portfolio. These choices related to the cabinet size are commonplace in Brazil, where the number of ministerial portfolios has oscillated from 9 up to 39 ministries. The Senate’s classification is a legal taxonomy that classifies and labels decrees by fields of law and their legal effects. Hence, it links decrees, as non-statutory decisions, to the Brazilian legal system. Since legal issues are cross-cutting to different ministries, we expect other clusters to emerge from our topic modeling strategies based on these two taxonomies.

**Fig 6:**
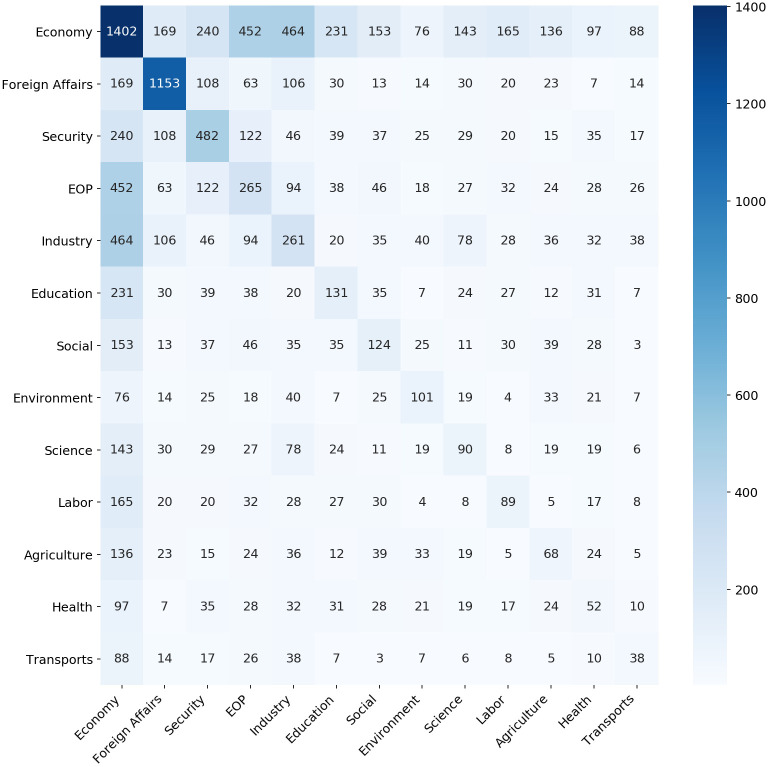
Co-sponsorship of cabinets’ policy-areas. The main diagonal stands for the number of decrees with only one label.

It is also usual to find decrees that affect one or more areas or are co-signed by two or more cabinets. Therefore, those classes are modeled in a multi-label schema. But how often does each area collaborate with another? Fig 6 presents a matrix illustrating how ministerial portfolios collaborate with each other while signing decrees. It is worth noting the central role played by the *EOP* group, which refers to the decisions made by the units of the Executive Office of the President, in the decree-making process. In support of the president’s decisions and inter-ministerial coordination, the *Economy* group plays a decisive gate-keeping power in the executive’s decrees. These units evaluate the economic and budgetary impact of the decrees, including those requested by other ministries. In consequence, these groups, *Economy* and *EOP*, tend to sign a large number of decrees together.

Notably, these patterns of co-authorship among ministries were also observed in the drafting of the Executive’s bill proposals before they were introduced to Congress. Rennó and Wojcik [39] note that the ministers of the Finance Ministry, Planning Ministry, Justice Ministry, Development, Industry and Foreign Trade Ministry, Labor Ministry, and Social Security Ministry co-signed, together with the president, the largest number of Executive Branch bill proposals from 1995 to 2010.

**Fig 7:**
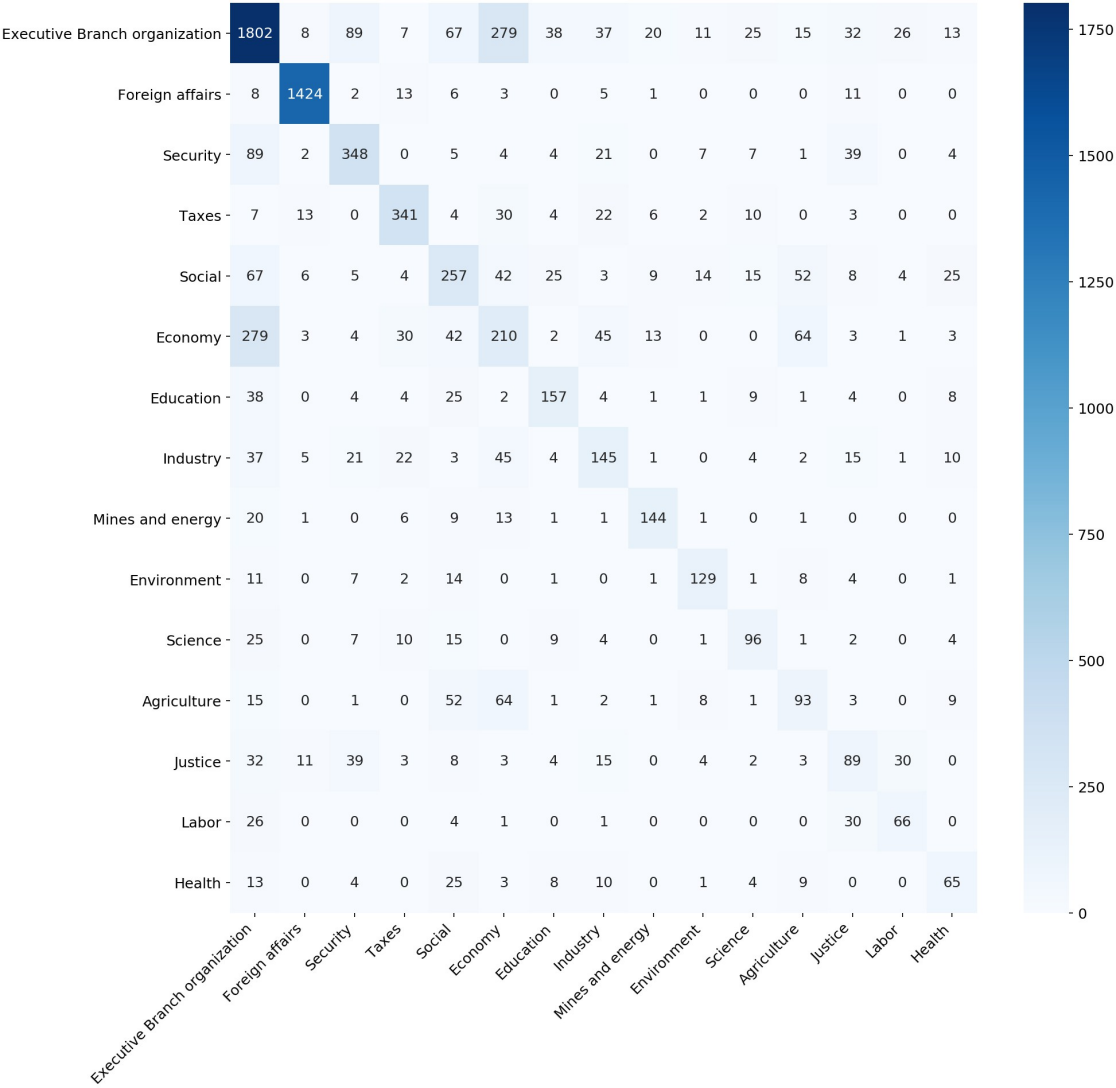
Collaboration between areas from the perspective of the Senate-based taxonomy. The main diagonal stands for the number of decrees with only one label.

While the pivotal role is notorious for our cabinets-based classification, the same is not observed in the Senate-based groups. Fig 7 illustrates the collaboration across fields of law. In that case, we observe that the class *Executive Branch organization* is the one that tends to collaborate with every other, while the *Economy* does not draw so much attention. This illustrates the differences between those two taxonomies and indicates that cabinet acts might go beyond the main areas of their portfolios.

While the pivotal roles of the *EOP* and *Economy* groups impact our cabinet-based classification, a different pattern emerges when we use the Senate-based groups. Fig 7 illustrates decrees labeled in one (main diagonal) or more fields of the law. In this case, we observe that the class *Executive Branch organization* is the one that tends to overlap with other classes, while the *Economy* class does not show broad transversality. It points out the differences between the two taxonomies we use in this analysis. It suggests that multi-labeling is more frequent in cabinets-based classification, because the ministries are in charge of different components of public policy (budget, administration, departmentalized bureaucracies) or depend on several executive agencies (sectoral, centralized and decentralized organs).

**Fig 8:**
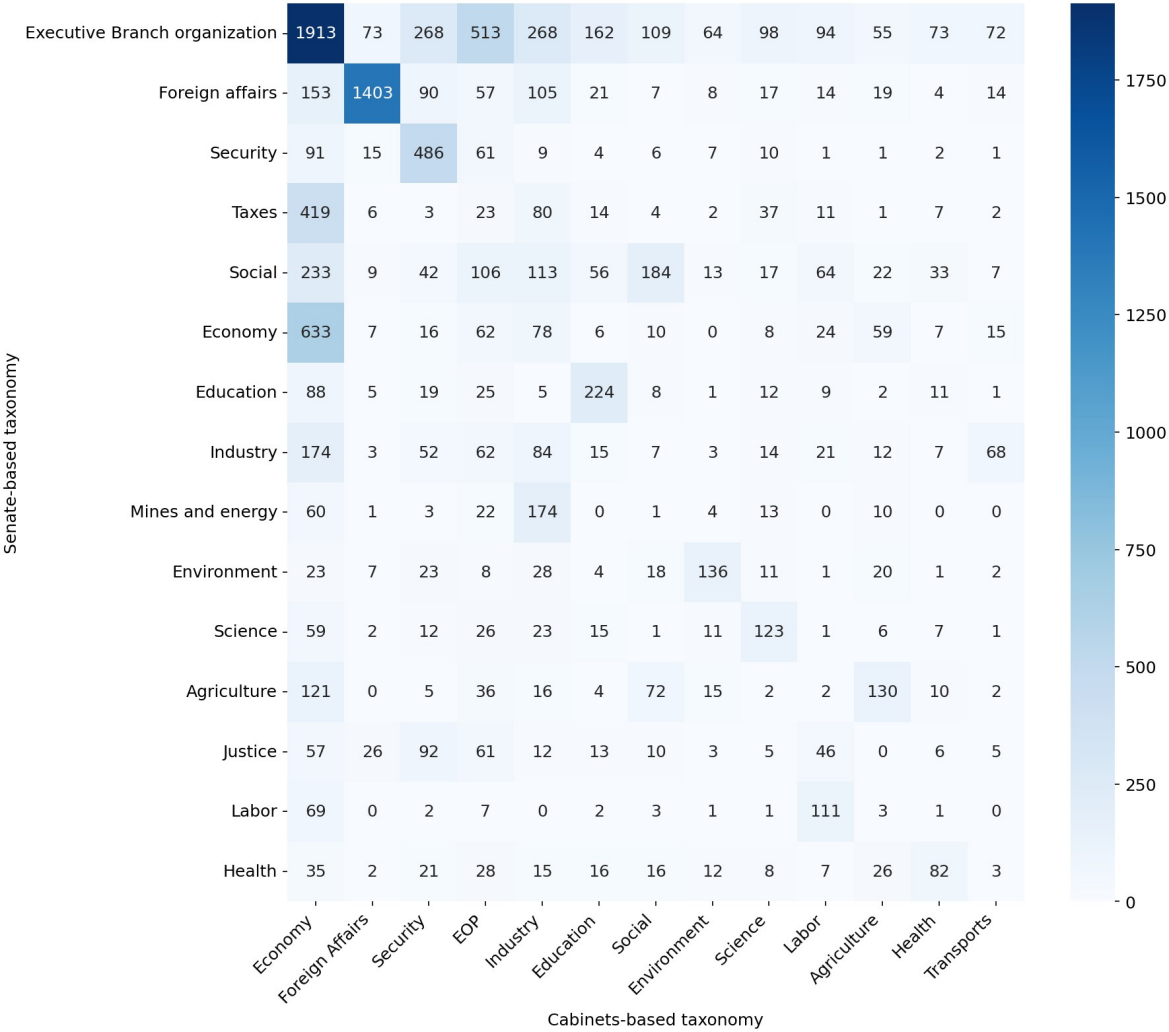
Overlap between the Senate and cabinets-based taxonomies.

Also, to explore how these two taxonomies overlap, Fig 8 shows the intersection between the classes of them. We highlight that some classes have similar descriptions and labels in both classifications, however, we have to bear in mind that these two taxonomies are based on conceptually distinct classifications of the contents of the decrees. Indeed some similar classes reveal consistency across taxonomies, e.g. *Foreign affairs*, *Security*, and *Education* (all homonyms in both taxonomies). However, the fields of law affect cross-cutting policies executed by different ministerial portfolios. In particular, *Industry* (note that this class also has a homonym in the cabinets-based taxonomy), *Taxes*, and *Executive Branch Organization* decrees from the perspective of the Senate-based classification tend to map to Cabinets related to the *Economy* class. This relates to the pivotal role of the ministries of Economy and Finance in Brazil and also illustrates how the characteristics explored by the taxonomies differ. The classification based on ministerial policy areas shows a more heterogeneous set of decisions, since cabinet units are more sensitive to political and administrative dynamics and their actions are hardly constrained to the fields of law.

Finally, it is worth noting that there are decrees unable to match any class: 165 for the Senate-based and 42 for the cabinets-based. We decided to remove those decrees from the dataset while performing the supervised text classification analysis, keeping only decrees classified by both taxonomies, i.e., 6,635 decrees.

#### Temporal analysis

**Fig 9:**
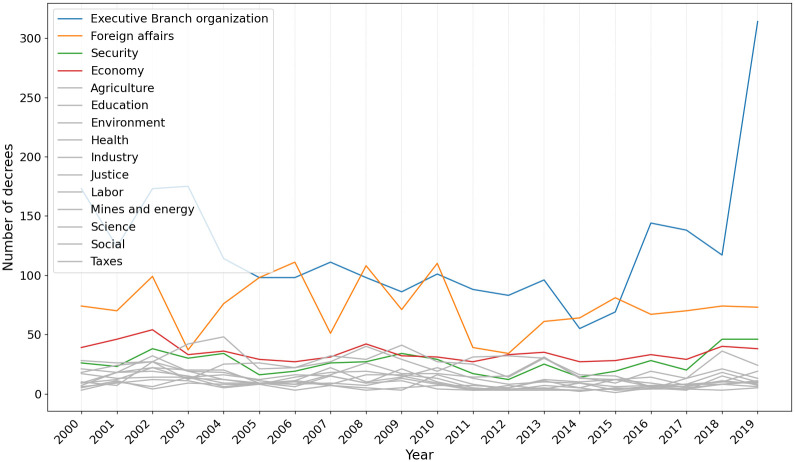
Number of decrees per year from the perspective of the Senate-based taxonomy focusing on the top 4 areas most issued in 2019.

**Fig 10:**
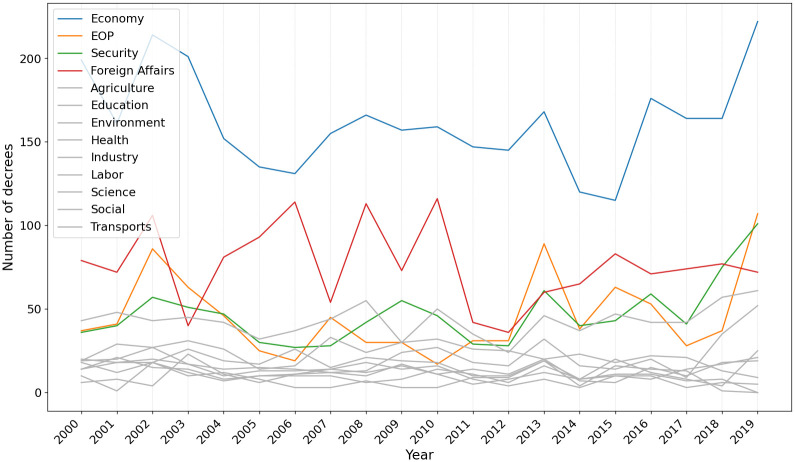
Number of decrees per year from the perspective of the cabinets-based taxonomy focusing on the top 4 areas most issued in 2019.

Taxonomies can also be used to infer the priorities of the Executive Branch across different administrations and situations. Figs 9 and 10 present the annual number of decrees related to each class respectively for the Senate and for the cabinets-based taxonomies.

From the Senate-based taxonomy perspective, most classes have decrees well distributed all over time. The number of decrees classified as *Executive Branch Organization*, however, peaked in 2019. This relates to the inauguration of the Bolsonaro’s administration in 2019, when the organization of the Federal Executive agencies has changed considerably.

The inter-temporal comparison of the number of decrees classified by the cabinet-based taxonomy shows interesting trends. First, economic issues concentrated the largest number of decrees issued by the president, despite slight fluctuations at some points in this time series. Second, the number of decrees in some classes has increased considerably since 2016. The number of decrees classified as *Economy*, *EOP*, and *Security* has peaked, reflecting constitutional and legal reforms approved by Congress and changes in public administration prioritized by the Temer (2016–2019) and Bolsonaro (2019-) governments. In particular, the advance in security decrees in Bolsonaro’s presidential term signals changes in the president’s policy priorities implemented administratively, not through new legislation. Therefore, this outcome is not captured by the Senate-based taxonomy. Finally, in the reverse direction, the number of decrees classified as *Foreign Affairs* has decreased since 2014.

### Supervised text classification

Table 6 presents the F1-score metric given by each of the approaches we have tested for the Senate-based classification. Regarding macro and micro averages, HAN is the single classifier that produces the best result on the test set; however, both macro and micro averages are quite similar to the ones of the SVM approach, which is a simpler and computationally cheaper method. It is worth noticing that some classes are accurately classified regardless of the used technique. This is the case for *Foreign Affairs* (F1-score always > 95%), *Taxes* (>90%), and *Security*, *Executive Branch organization*, *Mines and energy*, and *labor* (> 80%). Note that some of those areas relate to topics previously shown by the unsupervised analysis. Also, note that most results got better through majority voting, i.e., by combining the techniques. However, the combination of techniques requires the computation of all techniques over the dataset and, therefore, can be computationally expensive.

**Table 6:** F1-score for the Senate-based taxonomy models.

Class	SVM	CNN	HAN	Majority voting
Agriculture	0.790	0.757	**0.810**	0.795
Economy	0.815	0.762	**0.818**	0.845*
Education	0.713	**0.761**	0.729	0.769*
Environment	0.794	0.787	**0.857**	0.831
Executive Branch organization	0.870	0.865	**0.872**	0.886*
Foreign Affairs	**0.978**	0.972	0.976	0.978
Health	0.655	0.627	**0.721**	0.750*
Industry	0.685	0.538	**0.704**	0.654
Justice	**0.667**	0.500	0.659	0.667
Labor	**0.846**	0.807	0.815	0.873*
Mines and Energy	**0.917**	0.883	0.872	0.919*
Science	**0.862**	0.689	0.742	0.754
Security	0.852	**0.896**	0.882	0.903*
Social	0.758	0.777	**0.783**	0.812*
Taxes	0.916	0.908	**0.923**	0.929*
Macro F1-score	0.808	0.769	**0.811**	0.824*
Micro F1-score	0.855	0.840	**0.858**	0.873*

Results given according to the test set. Bold numbers along SVM, CNN, and HAN indicate the best results among single classifiers. Asterisks (*) on the majority voting column indicate classes for which results get better by combining the outputs of the three others classifiers.

We also highlight classes where all the supervised techniques tend to fail more. *Health*, *Industry*, and *Justice* are areas with this tendency when compared with the others in our database. In particular, *Justice* is the single class whose results were all under 70%. One reason that might explain this behavior for *Industry* and *Justice* is that those are classes that aggregate a vast set of different matters, that might also interfere in topics related to other classes.

Finally, results for the *Health* class can relate to the fact that, in Brazil, the most frequently used regulation to the health area is given through Ministerial Ordinances [40, 41], another type of textual decision issued by ministers on topics related to their respective policy area’s jurisdiction. Therefore, though some decrees are associated with this area, that is unlikely the most accurate tracking of *Health* decisions. A possible approach to improve results for this area would be enriching our training sets with samples from related Ministerial Ordinances to indicate better the particular terminologies and decisions of this area.

Table 7 presents F1-score for the cabinets-based taxonomy. Here, the Convolutional Neural Network outperforms the other two, indicating that no single approach is best for all taxonomies. Still, HAN and SVM approaches get close to those results on average and also outperform the Convolutional Neural Network in some particular classes. Some tendencies observed for the Senate-based classification can also be observed here. In particular, the performance of *Foreign Affairs* class is also greater than all other classes. Most of those results can again get better by ensembling the models through the majority voting system. However, we again emphasize the computational costs related to ensembles.

**Table 7:** F1-score for the cabinets-based taxonomy models.

Class	SVM	CNN	HAN	Majority Voting
Agriculture	0.737	**0.787**	0.710	0.739
Economy	0.876	0.886	**0.893**	0.909*
Education	**0.807**	0.785	0.795	0.788
Environment	0.765	**0.800**	0.780	0.840*
EOP	0.486	0.478	**0.508**	0.522*
Foreign Affairs	0.951	**0.958**	0.949	0.959*
Health	0.727	**0.744**	0.651	0.753*
Industry	0.719	0.730	**0.754**	0.760*
Labor	0.648	0.606	**0.707**	0.679
Science	0.730	0.722	**0.739**	0.771*
Security	0.758	**0.831**	0.777	0.818
Social	0.713	0.721	**0.741**	0.756*
Transports	0.679	0.764	**0.788**	0.821*
Macro F1	0.738	**0.755**	0.753	0.778*
Micro F1	0.794	**0.813**	0.808	0.830*

Results given according to the test set. Bold numbers along SVM, CNN, and HAN indicate the best results among single classifiers. Asterisks (*) on the majority voting column indicate classes for which results get better by combining the outputs of the three others classifiers.

We reassert that, though the idea of some areas in our two taxonomies is similar, the classification given for some samples can be pretty distinct, as the focus of each taxonomy is distinct. Still, we note that similar results are observed for some classes with similar descriptions to the ones of the Senate, for instance, the results given for *Health* class, and the already mentioned results for *Foreign Affairs*. Results for classes such as *Labor*, however, are notoriously different—in this particular case, worse—given the differences between the methodologies of each taxonomy.

The most notorious result relates to the *EOP* class, for which there is no similar class in the Senate-based taxonomy. This class is specifically related to the organization of the Presidency and decisions centralized by the Presidency’s units. Hence, it does not fit well in the Senate-based taxonomy. This class features the worst result, meaning all classifiers get results under 51%. This result can relate to the wide variety of topics, contexts, and situations that encompasses EOP. This, in turn, makes EOP classification based on textual features more challenging.

### Networks analysis

As stated previously, another question to be answered with the analysis of administrative decrees is: *Is it possible to identify priority policy issues or areas in each administration based on thematic clustering, co-authorship, and inter-connectivity of the decrees?* To answer that, it is worth noting that new laws or changes in presidents’ priorities lead to new decrees or the review of existing directives. Therefore, decrees commonly link previous directives or different policy areas in case of inter-ministerial decisions. Hence, by mapping these links, we can uncover the dynamics of the decree-making process in various regulatory areas and their impacts on policy outcomes. In this section, we have modeled our collection of decrees as a graph to explore further the intrinsic mechanisms related to Brazilian administrative decrees. Table 8 presents the main characteristics of this graph.

**Table 8:** Characteristics of the administrative decrees network.

Number of nodes	4489
Number of edges	7133
Average degree	1.58
Network Diameter	∞
Clustering coefficient	0.12
Number of nodes of the greatest connected component	3191
Number of edges of the greatest connected component	6064

**Fig 11:**
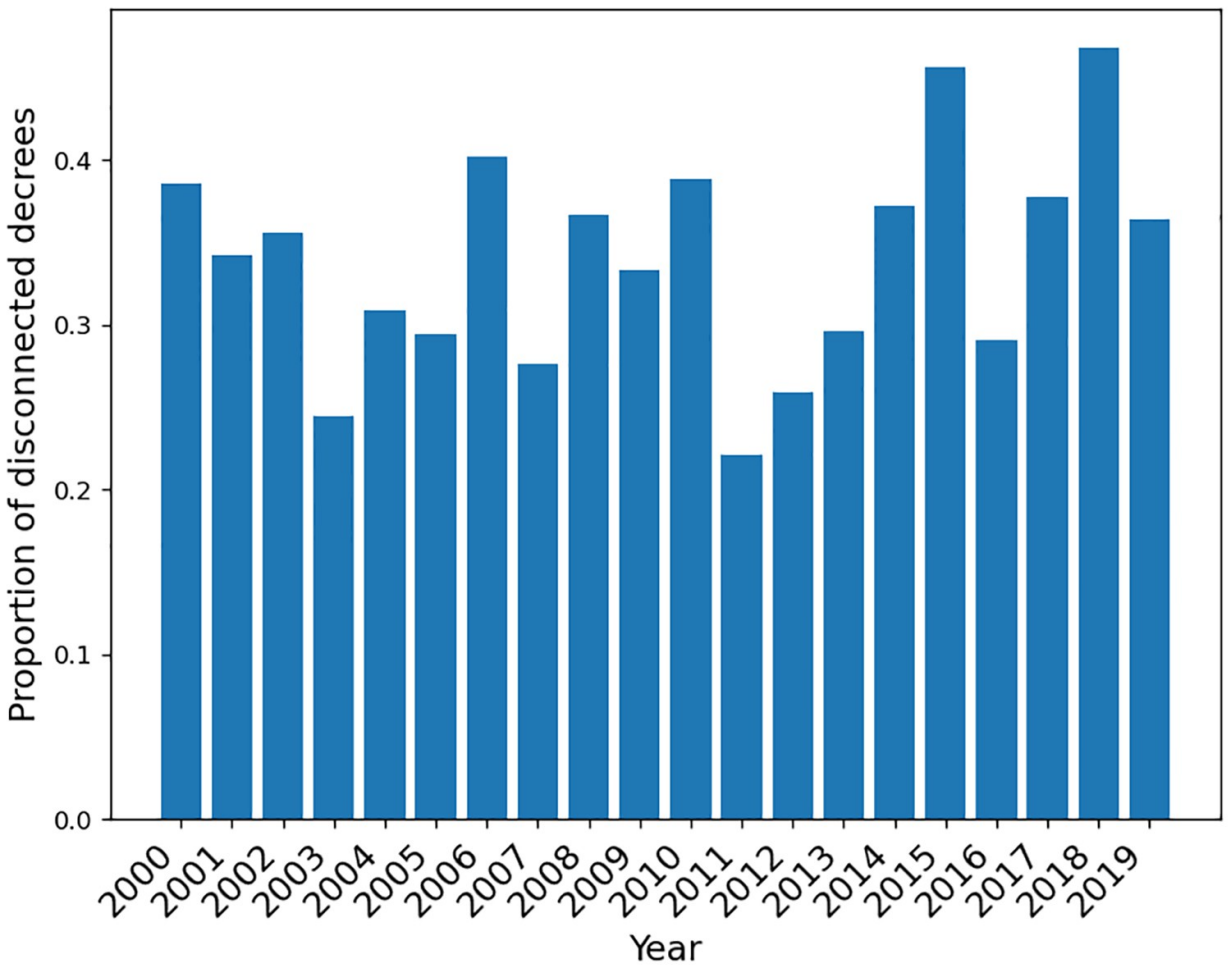
Disconnected decrees per year. Proportion of disconnected decrees given by (number of disconnected decrees)÷(total number of decrees in a year).

We note that 2,352 decrees of our 20-year-old collection (2000–2019) do not link to any other decree. We call them disconnected decrees and depict their proportion organized per year in Fig 11.

Note that the administration’s first years display a lower proportion of these disconnected decrees: 2003, 2007, 2011, 2016, and 2019. We believe that the number of disconnected decrees is so because the presidents’ first unilateral acts focus on revoking decrees from previous governments and on issuing decrees with decisions closer to their political preferences. The exception to this trend was in 2015 when the president resorts to a large proportion of disconnected decrees to cope with the political crisis and, after, to cope with the impeachment proceedings that paralyzed the administration from day one of the second presidential term of Dilma Roussef.

Last, per our analysis, decrees are disconnected if they do not relate to any other decree of our collection. Therefore, our set of disconnected decrees may include some related to other decrees issued outside the period 2000–2019. This abstraction, in turn, suggests that further analysis encompassing a more extended period is possible.

Next, we explore aspects of the modeled network from the microscopic and mesoscopic levels, like other analyses done for web links (e.g., [42]). S4 File give an indicative of the macroscopic structure of the disposition of administrative decrees online.

#### Microscopic properties of the administrative decrees’ network

Microscopic properties relate to individual nodes in a graph. Nevertheless, to present an overview of the most central elements of our network, we show in Table 9 the proportion of those nodes that relate to each of our taxonomies classes. By doing so, we aim to indicate which sorts of decrees carry the structure of the presented network and which areas play central roles. The analysis given by this table is based on a collection that includes only the 100 most central decrees according to each centrality. Varying the number of decrees in this analysis might lead to different perceptions of importance across areas that can be further explored by future works.

**Table 9:** Taxonomies’ 100 most central nodes.

Senate area	Degree	Betweenness	Cabinet area	Degree	Betweenness
Agriculture	0.0	0.0	Agriculture	1.0	3.0
Economy	27.0	1.0	Economy	**82.0**	**96.0**
Education	0.0	0.0	Education	0.0	5.0
Environment	1.0	0.0	Environment	2.0	1.0
Executive Branch organization	33.0	**88.0**	EOP	29.0	18.0
Foreign Affairs	3.0	1.0	Foreign Affairs	3.0	2.0
Health	0.0	0.0	Health	1.0	0.0
Industry	**41.0**	0.0	Industry	3.0	4.0
Justice	2.0	2.0	Labor	2.0	6.0
Labor	1.0	4.0	Science	2.0	5.0
Mines and Energy	2.0	0.0	Security	3.0	16.0
Science	0.0	1.0	Social	8.0	4.0
Security	0.0	0.0	Transports	1.0	0.0
Social	4.0	0.0	Unknown	0.0	0.0
Taxes	8.0	6.0	-	-	-
Unknown	4.0	2.0	-	-	-

The unknown class is given to nodes for which the taxonomy is not defined. Decrees with equal degrees sorted by out-degree. Row values do not add up to 100 due to decrees with more than one label. Those decrees count equally for each of their labels.

Per the Senate-based taxonomy, the top 100 most central decrees most relate to the industry. By individually analyzing these industry decrees, we observe that 44 of them relate to the PAC cluster (see section “Mesoscopic properties of the administrative decrees’ network”) due to its high density, which implies a high degree of centrality. This area is followed by *Executive Branch organization* and *Economy* classes. From the perspective of the cabinets-based taxonomy, the majority of those nodes relate to *Economy*. For betweenness centrality, based on the Senate, the majority relates to the *Executive Branch organization* area, while based on cabinets, most of them relates to *Economy*.

Table 10 gives the top 5 decrees according to the degree centrality, the centrality values, and the labels given by the Senate and cabinets-based taxonomies. Most of them are from 2019 and refer to decrees revoking many other decrees. Thus, those usually relate to the Executive Branch Organization from the perspective of the Senate-based taxonomy. From the perspective of the cabinets-based taxonomy, those relate mostly to EOP.

**Table 10:** Top 5 decrees according to degree centrality.

Decree	Degree	Senate areas	Cabinet areas
10.086/2019	135	Unknown*	EOP
6.944/2009	123	Executive Branch organization	Economy
10.179/2019	115	Unknown*	EOP
9.917/2019	97	Executive Branch organization	EOP
9.757/2019	81	Unknown*	EOP

* The unknown area is given to nodes for which the class was not originally defined due to broad tags in the Senate tagging system. Still, in this particular case, those could be considered *Executive Branch Organization* decrees.

Table 11 gives the same analysis according to betweenness centrality. In this case, the top decrees usually relate to positions and commissions, therefore those are usually classified as Executive Branch Organization and Economy. We also note that those decrees are older than the ones with greater degree-centrality. That relates to the fact that to increase betweenness centrality a decree should both cite and get cited by other decrees.

**Table 11:** Top 5 decrees according to betweenness centrality.

Decree	Betweenness	Senate areas	Cabinet areas
6.188/2007	0.000405	Executive Branch organization	Economy, EOP
7.462/2011	0.000333	Executive Branch organization	Economy, Science
6.944/2009	0.000235	Executive Branch organization	Economy
6.521/2008	0.000233	Executive Branch organization	Economy, EOP
5.783/2006	0.000218	Executive Branch organization	Economy

It is worth noticing that Economic-related ministerial portfolios, such as finance and planning, play an essential role in maintaining the network’s structure. Those are decrees related to the execution of economic plans, budgetary decisions, public investments, and others. This centrality of the economic area points to the internal hierarchies of the Executive structures, demonstrated here by the gatekeeper role of some of them exercised through presidential decrees.

#### Mesoscopic properties of the administrative decrees network

In this level of analysis, our network has gotten divided into 409 groups, indicating the presence of small groups with related decisions. We observe 29 communities of size (number of nodes) greater than 1% of the network (44 nodes) that are detailed and illustrated in S5 File.

Most of those large groups of decrees relate to *Executive Branch organization*, usually due to changes in its organizational units and regulations of the civil service. Still, it is also possible to observe highly homogeneous clusters related to *Foreign Affairs*, *Taxes*, and *Environment*. Most of those large groups relate to specific areas that are properly illustrated by our taxonomies. This observation indicates that the characteristics of this network take influence from the area and authorship of each administrative decree. Yet, some areas generate or influence several clusters, particularly, *Executive Branch organization* and *Economy*. Those clusters also have more links to others of similar composition.

One of the most engaging large clusters comprises a little more than 1% of the network’s nodes, but with more than 13% of the network’s edges. By checking the abstracts and texts of this cluster, we observe that this group relates to a major economic plan called Growth Acceleration Program—PAC (or Programa de Aceleração de Crescimento—PAC, in Portuguese). PAC execution took place during the presidents Silva’s and Rousseff’s terms, starting in 2007 and discontinued in 2014. This program required the intensive issuance of decrees to finance and execute public investments and programs in infrastructure, transportation, energy production and connectivity, and public housing throughout this period.

The influence of the PAC cluster has also been observed in the microscopic properties of the network, particularly in the degree-centrality analysis. Table 12 gives its full constitution regarding the Senate and cabinets taxonomies. It is possible to observe that the constitution of this cluster is also highly homogeneous from the perspective of both the taxonomies presented in this work.

**Table 12:** PAC cluster: Senate and cabinets taxonomies. Areas that are not present in this cluster were omitted.

Senate area	Number of decrees	Cabinet area	Number of decrees
Industry	80.00%	Economy	87.27%
Economy	45.45%	EOP	30.91%
Executive Branch organization	10.91%	Security	10.91%
Foreign Affairs	3.64%	Transports	3.64%
Labor	1.82%	Foreign Affairs	3.64%
Justice	1.82%	Labor	1.82%
-	-	Agriculture	1.82%
-	-	Science	1.82%

The presence of the PAC cluster can extend the perception of clusters created by particular areas, including the observation that specific regions of the network model might capture major economic plans handled by the Executive. We cannot verify whether this pattern also matches with older economic plans because of our data period. Yet, an interesting question is whether economic plans tend to create more dense regions in similar decisions networks. We encourage future works to explore this kind of group in the context of administrative decrees in Brazil and other countries. Notably, we believe that similar results can be found with various other methods for community analysis that can help tracking and uncovering groups of related decisions.

## Conclusion

This paper presented a framework for the analysis of administrative decrees in Brazil. We have collected decrees from 2000 to 2019, analyzed them using unsupervised learning, created taxonomies to describe their content from two perspectives, trained three supervised learning classifiers, and, finally, used those taxonomies to analyze the complex network generated by links between decrees.

During unsupervised analysis, we have observed textual patterns that supported the development of two taxonomies for the classification of administrative decrees. The former, based on the cabinets that sign each decree, focuses on policy areas. The latter, based on the Brazilian Senate’s tagging system, focuses on the fields of law of each decree. The design of taxonomies has shown great potential to understand and track the issuance of administrative decrees in Brazil, supporting most of the other results presented in this work.

Furthermore, we have automatically reproduced those classifications through supervised learning, in which our best classifier obtained more than 80% of macro F1-score. Results for classifications based on the Senate taxonomy tended to overperform results of classifiers based on cabinets. We highlight that the classification of administrative decrees is a task that manually takes great effort from different stakeholders of those decisions and that machine learning and NLP demonstrated promising results in automatically classifying those texts.

Using the taxonomies developed in this work, we have also identified variations in the issuance of administrative decrees during different administrations, with a peak of Executive Branch organization decrees in 2019, for example. Furthermore, we have shown which areas co-sponsor more decrees, observing the pivotal role of EOP and Economy from the perspective of the cabinets taxonomy and the essential role of the Executive Branch Organization from the perspective of the Senate taxonomy.

Finally, we have modeled the collection of administrative decrees as a graph and briefly analyzed it from the mesoscopic and microscopic levels. From the mesoscopic, we observed the potential of this kind of network of mapping different actions from the government, particularly notorious by the dense region created by the PAC program. From the microscopic level, we again observed the essential role of the economic-related areas as the ones that maintain the network structure.

The analysis of administrative decrees is a prominent field to track and understand decrees from time scopes other than 2000–2019. A future possibility, for example, is analyzing the effects of the Covid-19 pandemic in the Executive Branch actions through decrees: “Does these decrees create dense regions in the administrative decrees network?”, “What were the major areas involved?”. That analysis can also take great advantage of the Computer Science techniques applied in this work.

Other possible future works include exploring the performance of other classification approaches for texts from the Executive Branch in Brazil and Latin America, including transferred learning approaches. Also, it is possible to deepen the discussion on network analysis by labeling types of links and by exploring different centrality measures in this type of network.

## Supporting information

S1 File Brazilian administrative decree example. (PDF)

S2 File Supervised learning supporting information. (PDF)

S3 File Precision and recall detailed metrics. (PDF)

S4 File Considerations about the online web of decrees. (PDF)

S5 File Top modularity clusters of administrative decrees. (PDF)

S6 File English to Portuguese dictionary. (PDF)
