# Supplementary material for: The Executive Branch decisions in Brazil: A study of administrative decrees through machine learning and network analysis
Source: PLoS One. 2022 Jul 21;17(7):e0271741. doi: 10.1371/journal.pone.0271741 (PMC9302789; doi:10.1371/journal.pone.0271741)
Supplement: S1 File — (PDF) [file pone.0271741.s001.pdf]

# Supporting Information 1

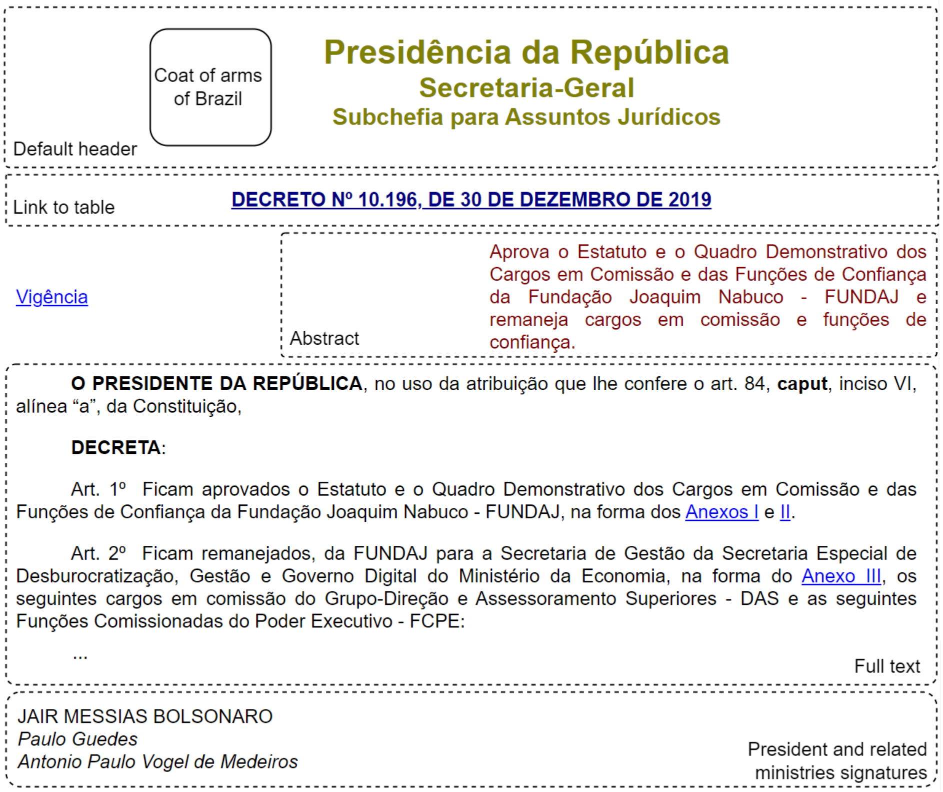


**Fig 1.** Example of a Brazilian administrative decree. Original document available at: http://www.planalto.gov.br/ccivil_03/_Ato2019-2022/2019/Decreto/D10196.htm. Accessed in November, 20, 2021.

**Table 1. Example of the table with the basic information given for a Brazilian administrative decree.**

| | |
|---|---|
| Signature date | December, 30, 2019 |
| Abstract | Approves the Bylaws and the Demonstrative Chart of Positions and functions of confidence of the Fundação Joaquim Nabuco - FUNDAJ and reassigns Positions and functions of confidence. |
| Situation | There is no express revocation. |
| Head of government | Jair Messias Bolsonaro |
| Origin | Executive Branch |
| Publishing date | December, 31, 2019 |
| Source | D.O.U. of 12/31/2019, p. 17 |
| Cabinets | Ministry of Economy - ME, Ministry of Education - MEC |
| Subjects | Approval, Administrative organization, Demonstrative chart, High-Level Management and Advisory positions (DAS), Commission position, Executive branch commissioned position (FCPE), Relocation, Extinction, Ambit, Joaquim Nabuco Foundation (FUNDAJ), Ministry of Economy. |
| Classification of laws | Executive Branch, Administrative Organization, Personnel. |

Original table (in Portuguese) available at: https://legislacao.presidencia.gov.br/atos/?tipo=DEC&numero=10196&ano=2019&ato=b2cc3Yq1keZpWTa45. Accessed in November, 20, 2021.
