# Supplementary material for: The Executive Branch decisions in Brazil: A study of administrative decrees through machine learning and network analysis
Source: PLoS One. 2022 Jul 21;17(7):e0271741. doi: 10.1371/journal.pone.0271741 (PMC9302789; doi:10.1371/journal.pone.0271741)
Supplement: S2 File — (PDF) [file pone.0271741.s002.pdf]

Supporting Information 2

As stated in this work, we have tested three individual approaches for supervised document classification. Further, we present the methodology given for each of them, including the pre-processing steps, the methodology for text representation, and the model itself.

As a baseline, we started by analyzing the performance of an SVM-based approach. As already discussed, SVMs have been successfully applied for document classification in many contexts and have long been one of the most prominent models for so. For this model, we have used a textual representation based on *tf-idf*. This procedure begins by representing each document as a bag of words, where the order and relations between words are not considered. Each word is then weighted by its *idf* in the collection and by its *tf* on the document. Particularly for this model, given the weaknesses of textual representations based on bag of words and the pipeline given in [1] for similar approaches, we have also lemmatized the terms of the collection to state the relation between words of the same linguistic root. Finally, we have trained a set of SVMs with linear kernel, combined based on the binary relevance method, to obtain a final classifier capable of dealing with the multi-label aspect of our problem.

As for neural networks, Fig 1 shows the architecture of both neural networks. For the convolutional neural network, we have used the model presented in [2] since it has been applied in Brazilian texts from another branch, the judiciary. Similar models have also been successfully used for sentence classification [3] and another CNN was applied for this problem on [4]. For this model, we have also standardized the size of documents in number of words, taking the 90% percentile of number of words as a threshold for crapping/padding.

In turn, the hierarchical neural network with attention mechanism (HAN) follows the same original architecture given in [5]. For this neural network, we also have standardized the size of the documents. As this architecture considers word and sentence levels, padding and crapping were applied both at sentence and document level, also in the 90% percentile.

Both approaches of neural networks used in this work presume the use of textual representations through embeddings. Due to the semantical properties of embeddings, for those models, we have opted not to apply lemmatization, as indicated in [6]. Also, the embedding layers' weights have been initialized based on a pre-trained model in Portuguese[1] [7]. Those weights are, however, also updated during the training procedure.

The final layer of both neural networks applies a sigmoid activation with the binary cross-entropy loss, compiled with the Adam optimizer [8]. To deal with imbalance, this loss is also weighted according to the representation of each class in our training dataset. However, this approach does not take into account the dependency between classes, and a more sophisticated method might be indicated in further experiments.

A final worst remark for the approaches based on neural networks relates to the non-determinism usually presented by the training procedure of those models. The nature of this process is not easily bypassed only by setting random seeds as it is in other methods such as the SVM implemented by Scikit Learn. However, determinism is essential while validating and analyzing a model. To deal with this issue, we have used the TensorFlow determinism library 0.3.0[2], a project supported by the Nvidia Corporation that implements determinism for TensorFlow. Still, results might vary depending on the machine and on the TensorFlow version. For the results presented in

[1] Glove Portuguese embeddings - 100 dimensions: http://www.nilc.icmc.usp.br/nilc/index.php/repositorio-de-word-embeddings-do-nilc. Accessed on November 20, 2021.

[2] TensorFlow Determinism Library: https://github.com/NVIDIA/framework-determinism. Accessed on November 20, 2021.

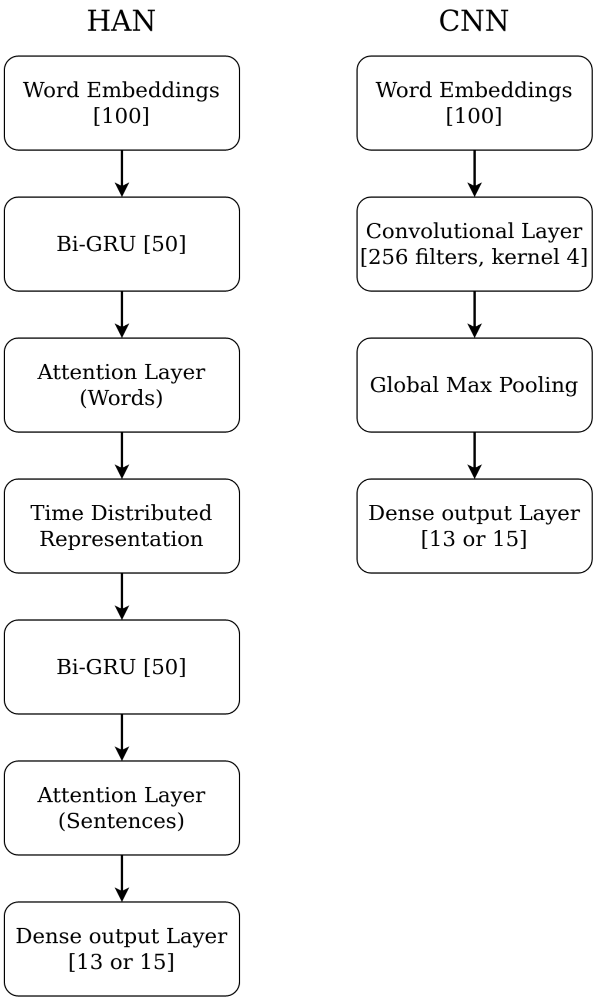

Fig 1. Architecture of the neural networks used in this work.

this work, all neural networks were trained in an Nvidia Quadro RTX 6000, with Cuda version 10.2 and TensorFlow version 2.0 running in the backend of the Keras library.

Finally, the hyper-parameters of each model were tuned based on a 4-fold approach. The final evaluation was performed in 20% of data initially discriminated. For the SVM, we have tuned the C of the model (Table 1), whereas, for neural networks, we have tuned the learning rate, number of epochs, batch size, beta1, and beta2 (Table 2). For that, we made use of Bayesian Optimization through the HyperMapper library [9], optimizing the macro F1-score metric. This metric was chosen given the unbalance between classes presented by our dataset. It is also worth noticing that we stratified all folds through [10], trying to maintain the original distribution, as can be observed in Fig 2 for the Senate and in Fig 3 for the cabinets-based taxonomy.

Table 1. SVMs' best hyper-parameters.

Model	C
SVM - Senate	15.397598
SVM - Cabinets	7.912664

Table 2. Neural networks' best hyper-parameters.

Model	Learning rate	Beta1	Beta2	Epochs	Batch Size
HAN - Senate	0.007347	0.802171	0.999	21.0	45.0
HAN - Cabinets	0.005434	0.77866	0.9999	11.0	61.0
CNN - Senate	0.00839	0.256272	0.999	11.0	55.0
CNN - Cabinets	0.003483	0.238497	0.9999	25.0	45.0

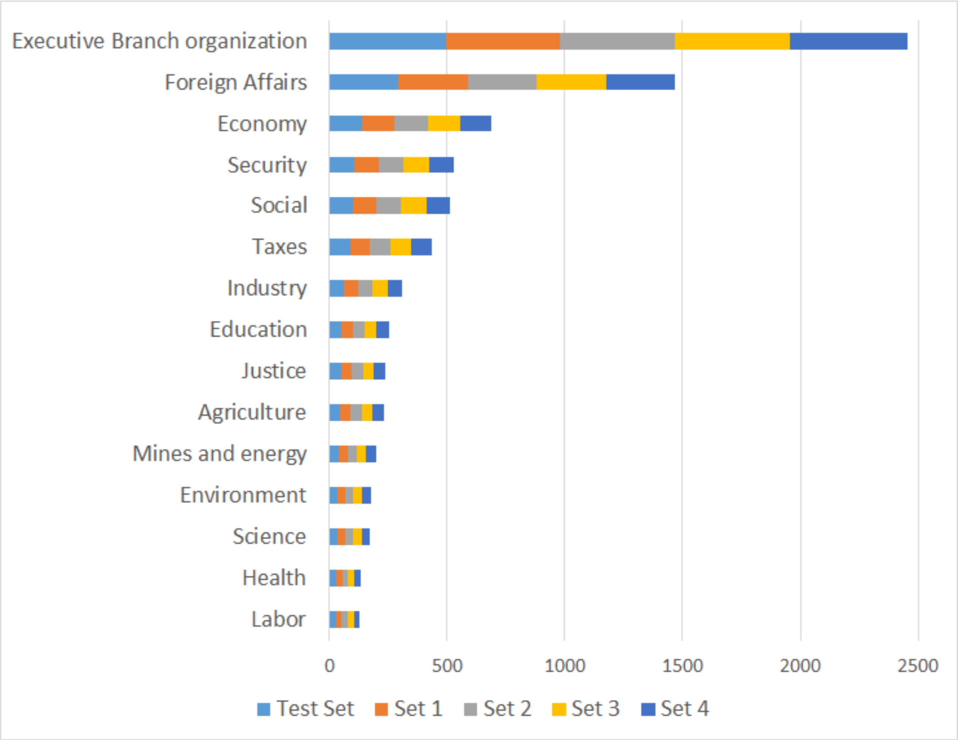

Fig 2. Distribution of labels for the Senate-based taxonomy.

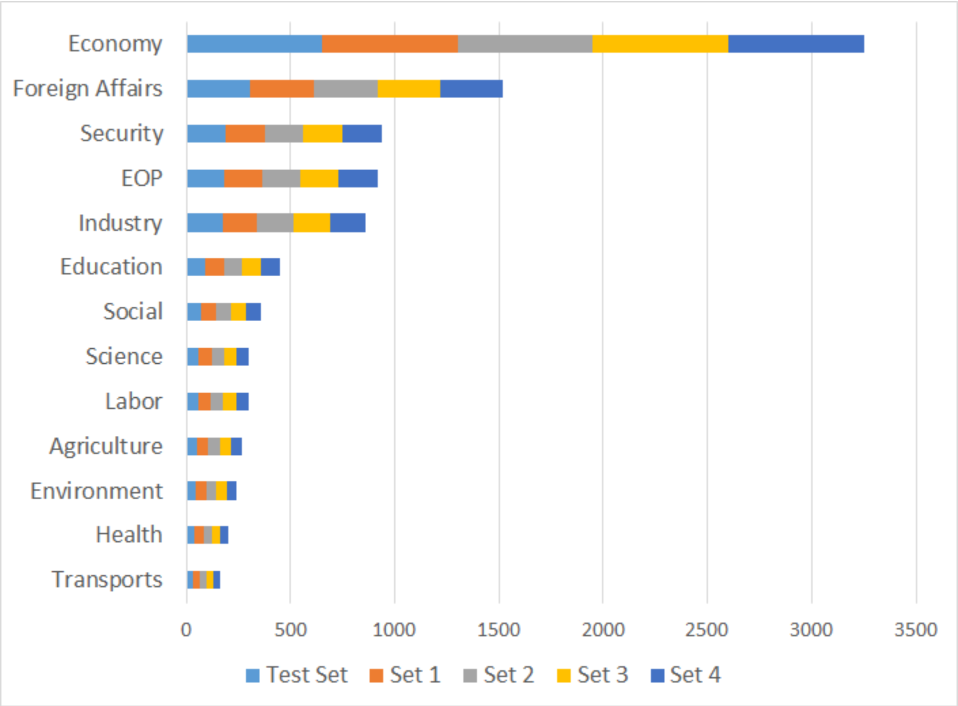

Fig 3. Distribution of labels for the Cabinets-based taxonomy.

References

1. Dalal MK, Zaveri MA. Automatic text classification: a technical review. International Journal of Computer Applications. 2011;28(2):37–40.

2. Da Silva NC, Braz F, de Campos T, Gusmao D, Chaves F, Mendes D, et al. Document type classification for Brazil's supreme court using a convolutional neural network. In: 10th International Conference on Forensic Computer Science and Cyber Law (ICoFCS), Sao Paulo, Brazil; 2018. p. 29–30.

3. Kim Y. Convolutional Neural Networks for Sentence Classification. In: Proceedings of the 2014 Conference on Empirical Methods in Natural Language Processing (EMNLP). Doha, Qatar: Association for Computational Linguistics; 2014. p. 1746–1751. Available from: `https://www.aclweb.org/anthology/D14-1181`.

4. Luz de Araujo PH, de Campos TE, Ataides Braz F, Correia da Silva N. VICTOR: a Dataset for Brazilian Legal Documents Classification. In: Proceedings of the 12th Language Resources and Evaluation Conference. Marseille, France: European Language Resources Association; 2020. p. 1449–1458. Available from: `https://www.aclweb.org/anthology/2020.lrec-1.181`.

5. Yang Z, Yang D, Dyer C, He X, Smola A, Hovy E. Hierarchical attention networks for document classification. In: Proceedings of the 2016 conference of the North American chapter of the association for computational linguistics: human language technologies; 2016. p. 1480–1489.

6. Camacho-Collados J, Pilehvar MT. On the role of text preprocessing in neural network architectures: An evaluation study on text categorization and sentiment analysis. arXiv preprint arXiv:170701780. 2017;.

7. Hartmann NS, Fonseca ER, Shulby CD, Treviso MV, Rodrigues JS, Aluísio SM. Portuguese Word Embeddings: Evaluating on Word Analogies and Natural Language Tasks. In: Anais do XI Simpósio Brasileiro de Tecnologia da Informação e da Linguagem Humana. Porto Alegre, RS, Brasil: SBC; 2017. p. 122–131. Available from: `https://sol.sbc.org.br/index.php/stil/article/view/4008`.

8. Kingma DP, Ba J. Adam: A method for stochastic optimization. arXiv preprint arXiv:14126980. 2014;.

9. Nardi L, Koeplinger D, Olukotun K. Practical design space exploration. In: 2019 IEEE 27th International Symposium on Modeling, Analysis, and Simulation of Computer and Telecommunication Systems (MASCOTS). IEEE; 2019. p. 347–358.

10. Sechidis K, Tsoumakas G, Vlahavas I. On the stratification of multi-label data. In: Joint European Conference on Machine Learning and Knowledge Discovery in Databases. Springer; 2011. p. 145–158.
