# Supplementary material for: The Executive Branch decisions in Brazil: A study of administrative decrees through machine learning and network analysis
Source: PLoS One. 2022 Jul 21;17(7):e0271741. doi: 10.1371/journal.pone.0271741 (PMC9302789; doi:10.1371/journal.pone.0271741)
Supplement: S3 File — (PDF) [file pone.0271741.s003.pdf]

Supporting Information 3

All the results here are given according to the test set. Bold numbers along SVM, CNN, and HAN indicate the best results among single classifiers. Asterisks (*) on the majority voting column indicate classes for which results get better by combining the outputs of the three others classifiers.

Table 1. Precision for the Senate-based taxonomy models.

Class	SVM	CNN	HAN	Majority voting
Agriculture	0.865	**0.933**	0.850	0.912
Economy	0.853	**0.903**	0.793	0.903
Education	**0.861**	0.854	0.778	0.875*
Environment	0.862	**0.889**	0.833	0.871
Executive Branch organization	0.885	**0.928**	0.908	0.925
Foreign Affairs	**0.993**	0.986	0.986	0.993
Health	0.679	**0.762**	0.710	0.808*
Industry	**0.760**	0.651	0.688	0.761*
Justice	**0.763**	0.704	0.714	0.844*
Labor	**0.957**	0.821	0.880	0.923
Mines and Energy	**0.971**	0.872	0.850	0.944
Science	**0.875**	0.750	0.793	0.821
Security	0.821	**0.928**	0.869	0.912
Social	0.750	0.811	**0.813**	0.830*
Taxes	0.962	**0.974**	0.951	0.963
Macro Precision	**0.857**	0.851	0.828	0.886*
Micro Precision	0.883	**0.907**	0.875	0.918*

Table 2. Recall for the Senate-based taxonomy models.

Class	SVM	CNN	HAN	Majority voting
Agriculture	0.727	0.636	**0.773**	0.705
Economy	0.780	0.660	**0.844**	0.794
Education	0.608	**0.686**	**0.686**	0.686
Environment	0.735	0.706	**0.882**	0.794
Executive Branch organization	**0.856**	0.810	0.838	0.850
Foreign Affairs	0.963	0.959	**0.966**	0.963
Health	0.633	0.533	**0.733**	0.700
Industry	0.623	0.459	**0.721**	0.574
Justice	0.592	0.388	**0.612**	0.551
Labor	0.759	**0.793**	0.759	0.828*
Mines and Energy	0.868	**0.895**	**0.895**	0.895
Science	**0.848**	0.636	0.697	0.697
Security	0.885	0.865	**0.894**	0.894
Social	**0.765**	0.745	0.755	0.796*
Taxes	0.874	0.851	**0.897**	0.897
Macro Recall	0.768	0.708	**0.797**	0.775
Micro Recall	0.829	0.781	**0.842**	0.832

Table 3. Precision for the Cabinets-based taxonomy models.

Class	SVM	CNN	HAN	Majority Voting
Agriculture	0.854	**0.925**	0.846	0.895
Economy	0.883	**0.948**	0.920	0.944
Education	**0.903**	0.865	0.889	0.887
Environment	0.886	0.872	**0.889**	0.971*
EOP	0.486	**0.610**	0.526	0.615*
Foreign Affairs	0.960	**0.983**	0.963	0.980
Health	0.800	**0.806**	0.659	0.829*
Industry	0.782	**0.810**	0.795	0.850*
Labor	0.714	**0.750**	0.719	0.766*
Science	0.764	**0.812**	0.804	0.857*
Security	0.780	**0.895**	0.801	0.873
Social	0.708	**0.863**	0.781	0.797
Transports	0.783	**0.840**	0.722	0.885
Macro Precision	0.793	**0.844**	0.793	0.858*
Micro Precision	0.821	**0.890**	0.840	0.890

Table 4. Precision for the Cabinets-based taxonomy models.

Class	SVM	CNN	HAN	Majority Voting
Agriculture	0.648	**0.685**	0.611	0.630
Economy	**0.868**	0.832	0.867	0.876*
Education	**0.730**	0.719	0.719	0.708
Environment	0.674	**0.739**	0.696	0.739
EOP	0.486	0.393	**0.492**	0.454
Foreign Affairs	**0.942**	0.935	0.935	0.938
Health	0.667	**0.690**	0.643	0.690
Industry	0.665	0.665	**0.717**	0.688
Labor	0.593	0.508	**0.695**	0.610
Science	**0.700**	0.650	0.683	0.700
Security	0.738	**0.775**	0.754	0.770
Social	**0.718**	0.620	0.704	0.718
Transports	0.600	0.700	**0.867**	0.767
Macro Recall	0.695	0.686	**0.722**	0.714
Micro Recall	0.769	0.747	**0.779**	0.777
