# Supplementary material for: The Executive Branch decisions in Brazil: A study of administrative decrees through machine learning and network analysis
Source: PLoS One. 2022 Jul 21;17(7):e0271741. doi: 10.1371/journal.pone.0271741 (PMC9302789; doi:10.1371/journal.pone.0271741)
Supplement: S4 File — (PDF) [file pone.0271741.s004.pdf]

Supporting Information 4

Considerations about the online web of decrees

Though during analysis we have modeled our collection as a citation network where an edge exists between two decrees if, and only if, the citing decree is newer than the cited one, it is worth noticing that by ignoring this condition on our data and analyzing the structure of links available online, our graph assumes a structure that can be well illustrated by the bow-tie decomposition. The bow-tie decomposition was reported on [1], firstly to represent the constitution of the graph of the web. Later on, it was also noticed on legal documents on [2].

On the web of the Planalto website, the presence of this structure in our collection is influenced by symmetric relations between citations. When a decree gets revoked or changed, it is usual to reference the change or revocation both on the cited decree and on the citing decree. That creates a Strongly Connected Component (SCC) that includes a substantial amount of our data and holds the bow-tie decomposition presented on Fig 1. This observation is mostly relevant in the context of information retrieval, particularly for specific domain web crawlers and search engines. Tubes were not observed. It is worth noticing that a great amount of decrees is included on the *Others* part, with small linked groups and isolated nodes. The amount of *Others* might also be related to the time scope of our data.

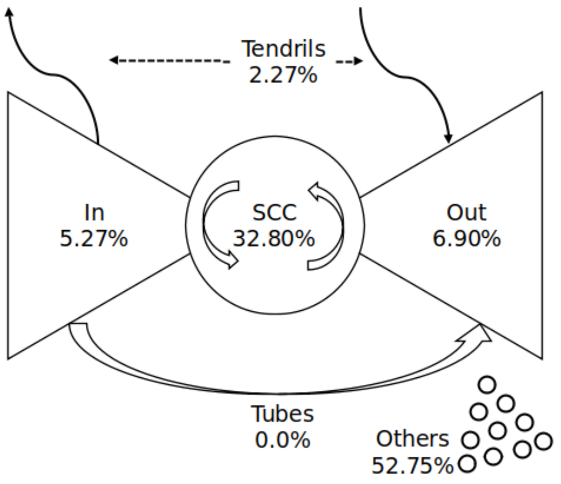

Fig 1. The bow-tie decomposition of the web of the decrees from 2000 to 2019.

References

1. Broder A, Kumar R, Maghoul F, Raghavan P, Rajagopalan S, Stata R, et al. Graph structure in the web. In: The Structure and Dynamics of Networks. Princeton University Press; 2011. p. 183–194.

2. Koniaris M, Anagnostopoulos I, Vassiliou Y. Network Analysis in the Legal Domain: A complex model for European Union legal sources. Journal of Complex Networks. 2018;6(2):243–268.
