# Supplementary material for: The Executive Branch decisions in Brazil: A study of administrative decrees through machine learning and network analysis
Source: PLoS One. 2022 Jul 21;17(7):e0271741. doi: 10.1371/journal.pone.0271741 (PMC9302789; doi:10.1371/journal.pone.0271741)
Supplement: S5 File — (PDF) [file pone.0271741.s005.pdf]

Supporting Information 5

Top modularity clusters of administrative decrees

Table 1. Top modularity communities' description.

C	N	E	Senate	Cabinets
1	221	303	EBO (72.85%), Ec (52.94%)	Ec (83.26%)
2	210	317	EBO (77.14%)	Ec (62.38%), EOP (53.33%)
3	198	334	Taxes (90.91%)	Ec (92.93%)
4	178	238	Social (43.26%), EBO (21.35%)	Social (39.89%), Ec (35.39%)
5	177	212	EBO (48.02%)	Ec (60.45%)
6	128	201	EBO (61.72%), Ec (50.00%)	Ec (89.84%)
7	128	261	EBO (90.62%)	Ec (92.19%)
8	127	166	EBO (36.22%)	Ec (86.61%)
9	117	339	Ec (33.33%), EBO (26.50%), Sec (24.79%)	Ec (47.86%), Sec (25.64%), Agri (20.51%)
10	108	144	EBO (62.96%)	Ec (68.52%)
11	103	134	EBO (72.82%)	Ec (56.31%), EOP (29.13%), Science (29.13%)
12	102	142	EBO (78.43%), Labor (22.55%)	Ec (83.33%), Industry (36.27%), Labor (26.47%)
13	99	135	Social (47.47%)	Ec (54.55%), Industry (52.53%), EOP (26.26%)
14	91	130	EBO (53.85%), Sec (37.36%)	Sec (62.64%), Ec (57.14%), EOP (21.98%)
15	84	133	EBO (60.71%)	Sec (69.05%), Ec (51.19%), EOP (22.62%)
16	81	104	Env (60.49%)	Env (79.01%), Industry (24.69%), Ec (23.46%)
17	78	117	Sec (28.21%) EBO (23.08%)	EOP (29.49%), Sec (25.64%)
18	74	74	Foreign Affairs (100.00%)	Foreign Affairs (100.00%)
19	64	87	Sec (71.88%), EBO (56.25%)	Sec (79.69%), Ec (54.69%)
20	62	96	Mines and Energy (98.39%)	Industry (98.39%)
21	59	85	EBO (89.83%)	Ec (77.97%), Ed (45.76%)
22	57	72	Ed (49.12%), Social (31.58%), EBO (31.58%)	Ed (59.65%), Social (40.35%), Ec (38.60%)
23	55	967	Industry (80.00%), Ec (45.45%)	Ec (87.27%), EOP (30.91%)
24	55	66	EBO (98.18%)	Ec (94.55%)
25	53	59	Agri (37.74%), Health (32.08%), EBO (22.64%)	Agri (83.02%), Ec (24.53%)
26	53	64	EBO (84.91%)	Ec (94.34%), Industry (22.64%)
27	50	61	EBO (94.00%)	EOP (48.00%), Ec (46.00%), Foreign Affairs (40.0%)
28	49	73	Taxes (97.96%), Ec (30.61%)	Ec (100.00%)
29	45	52	EBO (71.11%)	Ec (86.67%), EOP (26.67%)

C: Community id. N: Number of nodes. E: number of edges. EBO: Executive Branch Organization. Sec: Security. Agri: Agriculture. Ec: Economy. Ed: Education. Env: Environment
Groups are sorted according to the number of nodes. Only the top 3 classes with at least 20% of representativity are shown for better visualization. Percentages can add up to over 100% due to the intersections in the multi-label classifications.

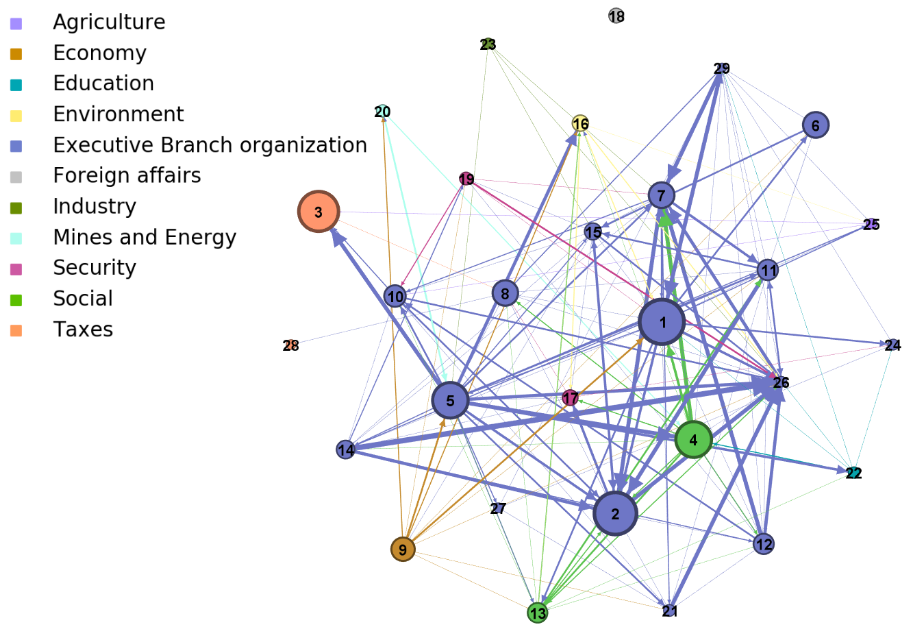

Fig 1. Main modularity clusters. Each node represents a cluster, with the size proportional to the number of decrees in it. The colors relate to the predominant area given by the Senate-based taxonomy. Finally, the weight of each edge is proportional to the absolute number of edges between clusters.
