# Supplementary material for: The Executive Branch decisions in Brazil: A study of administrative decrees through machine learning and network analysis
Source: PLoS One. 2022 Jul 21;17(7):e0271741. doi: 10.1371/journal.pone.0271741 (PMC9302789; doi:10.1371/journal.pone.0271741)
Supplement: S6 File — (PDF) [file pone.0271741.s006.pdf]

Supporting Information 6

English to Portuguese dictionary

This paper considers the following translations for Brazilian Portuguese words in our data:

- activities: atividades
- additional: adicional
- administration: administração
- advisory: assessoramento
- aeronautical: aeronáutico
- agency: órgão
- agrarian: agrário
- agree: acordar
- agrochemical: agrotóxicos
- aliquot: alíquota
- alter: alterar
- alternate: suplente
- ambit: âmbito
- animal: animal
- annex: anexar
- apostille: apostilamentos
- appropriation: dotação
- area: área
- arm: armar
- assistance: assistência
- bidding: licitação
- body: corpo
- bonus: gratificação
- budget: orçamentária
- camex: camex
- capital: capital
- chamber: câmara
- child: criança
- civilian: civil
- collection: arrecadação
- commission: comissão
- committee: comissão
- common: comum
- competence: competência
- complementation: complementação
- concessionaire: concessionário
- confer: conferir
- congress: congresso
- contracting: contratação
- control: controlo
- convention: convenção
- cooperation: cooperação
- coordinator: cordenador
- corporate: empresar
- corruption: corrupção
- council: conselho
- course: curso
- credit: crédito
- cultura: Culture
- customs: aduaneiro
- deadline: prazo
- december: dezembro
- decision: decisão
- development: desenvolvimento
- drafting: redação
- economic: econômica
- edict: edital
- education allowance: Salário educação

- education plan: Plano de educação
- education: educação
- electric: elétrica
- enact: decretar
- energy: energia
- environment: Meio ambiente
- ethical: ético
- evaluation: avaliação
- executive: executivo
- expense: despesa, dispêndio
- facility: instalação
- family: familiar
- federal: federal, federar
- federative: federativo
- financial: financeiro
- fire: fogo
- fiscal year: exercício
- follow: seguir
- forum: fórum
- frame: quadrar
- function of confidence: confiança
- function: função
- functioning: funcionamento
- go to: ir
- goods: mercadoria
- govern: governar
- grape: uva
- greeting: saudar
- group: grupar, grupo
- growth: crescimento
- health: Saúde
- human: humano
- hypothesis: hipótese
- implementation: implementação
- import: alíquota
- impose: impor
- incidence: incidência
- incident: incidente
- inclusion: inclusão
- individual: individual
- information: informação
- innovation: inovação
- inss: inss
- institution: instituição
- institutional: institucional
- interministerial meeting: interministerial
- interministerial: interministerial
- international: internacional
- january: janeiro
- law: direito
- law: lei
- legal: jurídico
- libre: libério
- line: linha
- majority: maioria
- maritime: marítimo
- may: maio
- medal: medalhar
- meeting: reunião
- member: membro
- merit: mérito
- military: militar
- Military Command: Comando militar
- Ministry of Communications of the Presidency: Secretaria de Comunicação Social da Presidência da República

- Ministry of Defense: Ministério da Defesa
- Ministry of Economy - ME: Ministério da Economia - ME
- Ministry of Education - MEC: Ministério da Educação - MEC
- Ministry of Foreign Affairs: Ministério das Relações Exteriores
- Ministry of Justice and Public Security: Ministério da Justiça e Segurança Pública
- Ministry of Social Development : Ministério do Desenvolvimento Social
- Ministry of Women, Family, and Human Rights: Ministério da Mulher, da Família e dos Direitos Humanos
- national: nacional
- naval: naval
- new: novo
- october: outubro
- official: oficial
- officiate: oficiar
- operation: operação
- packaging: embalagem
- parliament: plenário
- participation: participação
- perform: desempenhar
- police: polícia
- political: político
- port: porto, portuário
- position: cargo
- preference: preferência
- presidency: presidência
- president: presidente
- price: preço
- privatization: desestatização
- product: produto
- professional: profissional
- programming: programação
- promotion: promoção
- property: imóvel
- protocol: protocolo
- provide: ministrar
- providence: providência
- provision: dispositivo, providência
- public health: Saúde pública
- public servant: servidor
- public: público
- public: público
- racial: racial
- rate: importação
- reform: reformar
- regimen: regime
- regimental: regimental
- regulate: regulamentar
- relevant: relevante
- representative: representante
- republic: repúblico
- request: requerimento
- research: pesquisar
- resolution: resolução
- review: revisão
- rural: rural
- scientific: científico
- score: pontuação
- secretariat: secretariar
- Secretariat of Strategic Affairs of the Presidency: Secretaria de Assuntos Estratégicos da Presidência da República

- security: segurança
- segurança: Public Security
- senior: idoso
- september: setembro
- settlement: liquidação
- social security: previdência
- social: social
- society: sociedade
- state: estatal
- strategic: estratégico
- structure: estruturar
- superintendence: superintendência
- superior: superior
- supply: abastecimento
- sustainable: sustentável
- tax: constribuição, taxar
- teach: ensinar
- technological: tecnológico
- technology: tecnologia
- tecnical: técnico
- temer: Temer(presidente)
- thematic: temático
- time: tempo
- tourist: turístico
- trade: comércio
- transgenic Product: Produto transgênico
- transmission: transmissão
- trust: confiança
- undertaking: empreendimento
- universalization: universalização
- validity: vigência
- value: valor
- wine: vinho
- work: trabalhar
- writing: redação
